# Reentrant liquid condensate phase of proteins is stabilized by hydrophobic and non-ionic interactions

**DOI:** 10.1038/s41467-021-21181-9

**Published:** 2021-02-17

**Authors:** Georg Krainer, Timothy J. Welsh, Jerelle A. Joseph, Jorge R. Espinosa, Sina Wittmann, Ella de Csilléry, Akshay Sridhar, Zenon Toprakcioglu, Giedre Gudiškytė, Magdalena A. Czekalska, William E. Arter, Jordina Guillén-Boixet, Titus M. Franzmann, Seema Qamar, Peter St George-Hyslop, Anthony A. Hyman, Rosana Collepardo-Guevara, Simon Alberti, Tuomas P. J. Knowles

**Affiliations:** 1 Centre for Misfolding Diseases, Yusuf Hamied Department of Chemistry, University of Cambridge, Cambridge, UK; 2 Cavendish Laboratory, Department of Physics, University of Cambridge, J J Thomson Avenue, Cambridge, UK; 3 Department of Genetics, University of Cambridge, Cambridge, UK; 4 Yusuf Hamied Department of Chemistry, University of Cambridge, Lensfield Road, Cambridge, UK; 5 Max Planck Institute of Molecular Cell Biology and Genetics (MPI-CBG), Dresden, Germany; 6 Biotechnology Center (BIOTEC), Center for Molecular and Cellular Bioengineering (CMCB), Technische Universität Dresden, Tatzberg 47/49, Dresden, Germany; 7 Institute of Physical Chemistry, Polish Academy of Sciences, Kasprzaka, 44/52 01-224 Warsaw, Poland; 8 Cambridge Institute for Medical Research, Department of Clinical Neurosciences, University of Cambridge, Cambridge, UK; 9 Division of Neurology, Department of Medicine, University of Toronto and University Health Network, Toronto, Ontario Canada

**Keywords:** Proteins, Biophysics, Intrinsically disordered proteins, Biophysical chemistry, Biological physics

## Abstract

Liquid–liquid phase separation of proteins underpins the formation of membraneless compartments in living cells. Elucidating the molecular driving forces underlying protein phase transitions is therefore a key objective for understanding biological function and malfunction. Here we show that cellular proteins, which form condensates at low salt concentrations, including FUS, TDP-43, Brd4, Sox2, and Annexin A11, can reenter a phase-separated regime at high salt concentrations. By bringing together experiments and simulations, we demonstrate that this reentrant phase transition in the high-salt regime is driven by hydrophobic and non-ionic interactions, and is mechanistically distinct from the low-salt regime, where condensates are additionally stabilized by electrostatic forces. Our work thus sheds light on the cooperation of hydrophobic and non-ionic interactions as general driving forces in the condensation process, with important implications for aberrant function, druggability, and material properties of biomolecular condensates.

## Abstract (web summary)

Elucidating the molecular driving forces underlying liquid–liquid phase separation is a key objective for understanding biological function and malfunction. Here the authors show that a wide range of cellular proteins, including FUS, TDP-43, Brd4, Sox2, and Annexin A11, which form condensates at low salt concentrations, can reenter a phase-separated regime at high salt concentrations.

## Introduction

Liquid–liquid phase separation (LLPS) has emerged as an important organizing principle in biology, where condensation of proteins and other biomolecules into liquid droplets has been shown to underlie the formation of membraneless subcellular compartments^1–3^. Beyond compartmentalization, these biomolecular condensates have been implicated in diverse biological processes, including chromatin reorganization^4^, noise buffering^5^, and sensing^6^, and their misregulation has been associated with the emergence of diverse pathologies, such as neurodegenerative diseases and cancer^7–9^.

LLPS is a thermodynamic process in which weakly interacting proteins, and in many cases oligonucleotides, minimize their free energy by demixing into a protein-depleted dilute phase and a protein-enriched condensed phase^10–12^. LLPS often becomes thermodynamically favorable at biomolecular concentrations and under solution conditions where the free-energy gain from the dynamic formation of weak attractive intermolecular interactions^13^, and the increase in entropy associated with the release of water molecules from the surfaces of biomolecules to the bulk phase^14^, become sufficient to overcome the entropy loss due to the reduction in available solute microstates upon demixing^10–12^. This intricate balance of entropic and enthalpic forces raises the fundamental question about the nature of the molecular interactions that govern protein LLPS and of the factors that modulate them.

Previous experimental and theoretical studies have shed light on the molecular grammar underlying LLPS^15–17^. Accordingly, protein condensation has been shown to be driven by the action of both electrostatic and hydrophobic interactions, including charge–charge, cation–π, dipole–dipole, and π–π stacking interactions. The interplay of these interactions underlies the phase separation behavior of proteins at or below physiological ionic strength.

Here, we show that a wide range of proteins, including fused in sarcoma (FUS)^18–22^, transactive response DNA-binding protein of 43 kDa (TDP-43)^23,24^, Bromodomain-containing protein 4 (Brd4)^25,26^, sex-determining region Y-box 2 (Sox2)^27^, and annexin A11 (A11)^28^, which are known to undergo LLPS via homotypic multivalent interactions at low-salt concentrations, can also undergo LLPS at a high-salt concentration (i.e., above 1.5 M NaCl), reentering into a phase-separated regime from a well-mixed state at intermediate salt concentrations. This type of reentrant phase behavior—i.e., where the monotonic variation of a single thermodynamic control parameter drives proteins from a phase-separated state to a macroscopically similar state via two-phase transitions^29^—is in contrast to the established RNA-mediated reentrant behavior of protein–RNA coacervates assembled through heterotypic multivalent interactions, which are stable only in the presence of intermediate RNA concentrations, but exist as homogeneous solutions at both high and low RNA concentrations^30–33^.

LLPS at high-salt concentrations has been observed for a few polymer systems and proteins, but only at very high polymer/protein concentrations, typically in the hundreds of micromolar to the millimolar range, low temperatures, and/or extremes of pH^34–43^. Importantly, the reentrant protein LLPS we report here takes place at the low micromolar protein concentrations, temperatures, and pH values typical of physiological LLPS. While occurring at salt concentrations higher than those present physiologically (i.e., at ~2–3 M), the observation of an additional phase transition at high-salt underscores the complexity of the dynamic processes that underlie condensate formation and dissolution^1–3^, and the factors that control them, such as changes in scaffold concentration^44,45^, fluctuations in the condensate environment^46,47^, and many others^32^.

Crucially, our data reveal that the molecular interactions stabilizing condensates in the high-salt reentrant regime are fundamentally distinct from those driving phase separation at low salt. At high-salt concentrations, LLPS is mainly driven by hydrophobic and nonionic interactions. This ability of salt to shift completely the molecular driving forces of protein LLPS is consistent with the wide body of work demonstrating the significance of salt in the modulation of protein stability^48–51^, protein solubility^52,53^, protein–protein interactions^54,55^, and protein–nucleic acid interactions^56–58^.

Hence, our work demonstrates that the preferential interactions that the different amino acids establish in LLPS are heavily context-specific (i.e., they are defined not only by the amino acid chemical makeup but also by the microenvironment and conditions they are exposed to). For example, we show that some amino acid pairs transition from establishing dominant electrostatic attraction or repulsion to engaging instead in strong hydrophobic attraction, as a function of the salt concentration.

As such, this dominant role of hydrophobicity and nonionic interactions in the high-salt regime expands the molecular grammar governing LLPS, and demonstrates that the driving forces for protein phase separation are not only dictated by the amino acid sequence but also by the condensate environment. Overall, these findings may have wide-ranging implications for the interactions, druggability, and material properties of biomolecular condensates, and thus broaden our understanding of biomolecular condensate behavior in health and disease.

## Results

### Salt-mediated reentrant phase separation of FUS and other phase-separating proteins

**Fig. 1:**
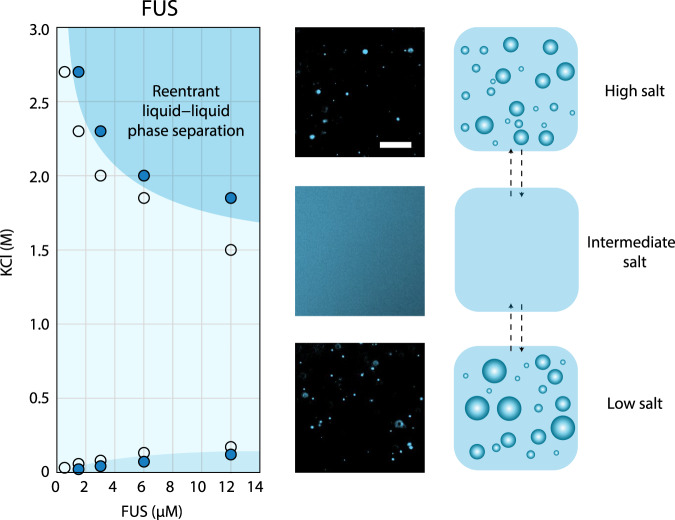
Reentrant phase separation of FUS at high salt. Phase diagram (left), representative images (center), and schematic (right) of FUS phase separation in the presence of increasing concentrations of KCl. In the phase diagram, markers filled with blue indicate concentrations where phase separation was observed in fluorescence images. Open markers indicate concentrations tested where phase separation did not occur. Darker blue regions are guides for the eyes indicating regions where phase separation of FUS occurs, and light blue is the region where no phase separation occurs. The reentrant phase separation regime is indicated. Fluorescent images of FUS (6 μM, EGFP labeled) were taken at 50 mM (low salt), 500 mM (intermediate salt), and 2.7 M KCl (high salt) in 50 mM Tris-HCl (pH 7.2). Scale bar is 20 µm. Each data image (center panel) is representative of the observed behavior from at least three test replicates of the respective protein/salt conditions.

We first discovered reentrant phase behavior for the protein FUS, when mapping out its phase diagram as a function of KCl concentration (Fig. 1). FUS remains phase-separated in salt concentrations up to ~125 mM, in line with previous observations^18–22^, and then forms a well-mixed phase between 125 mM and 1.5 M, but surprisingly reenters the phase-separated regime above 1.5 M KCl. Hence, FUS exhibits two-phase boundaries, at respective upper and lower transition concentrations of salt. Importantly, condensate formation at high KCl concentrations is fully reversible. Adjusting the KCl concentration back to the 500 mM to 1.5 M range yields a well-mixed phase, which is also known to occur for condensates at low-salt conditions upon increasing KCl concentration^21^.

**Fig. 2:**
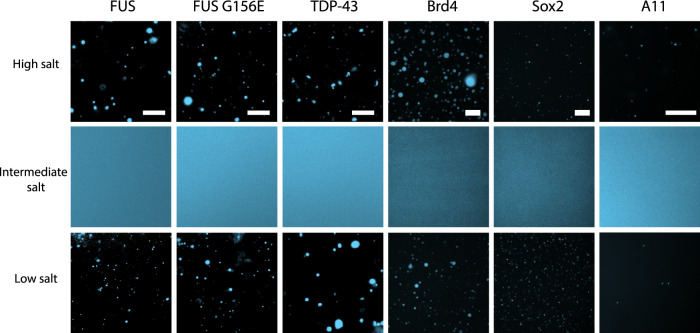
Salt-mediated reentrant phase separation of FUS, FUS G156E, TDP-43, Brd4, Sox2, and A11. Representative images of FUS, FUS G156E, TDP-43 at 50 mM (low salt), 500 mM (intermediate salt), and 2.7 M KCl (high salt) in 50 mM Tris-HCl (pH 7.2); Brd4 and Sox2 at 50 mM (low salt), 500 mM (intermediate salt), and 2.15 M KCl (high salt); Brd4 buffer: 5 mM Tris (pH 7.5), 0.2 mM EDTA, 0.5% glycerol; Sox2 buffer: 5 mM Bis-Tris-Propane (pH 7.5), 0.5% glycerol; and A11 at 22.5 mM (low salt), 225 mM (intermediate salt), and 500 mM NaCl (high salt) in 20 mM HEPES (pH 7.0). For fluorescence imaging, both FUS variants and TDP-43 were tagged with EGFP and studied at a protein concentration of 6 μM; Brd4 and Sox2 were tagged with monoGFP and studied at protein concentrations of 6 µM and 12.4 µM, respectively; A11 was labeled with AlexaFluor647 and studied at a protein concentration of 15 μM. Scale bars are 20 µm in all images. Each data image (center panel) is representative of the observed behavior from at least three test replicates of the respective protein/salt conditions.

In addition to FUS, we find salt-induced reentrant phase behavior in the pathological G156E mutant of FUS^18,19^, TDP-43^23,24^, Brd4^25,26^, Sox2^27^, and A11^28^ (Fig. 2). Like FUS, all these proteins phase-separate via homotypic multivalent interactions at low salt, are involved in essential cellular and developmental processes^59,60^, and implicated in neurodegenerative disorders^7,28^. Notably, in all cases, high-salt condensates have similar size distributions and shapes as their low-salt counterparts. An analysis of droplet shape revealed that both low- and high-salt condensates exhibit a high degree of circularity (>95%), and have similar areal distributions (Supplementary Fig. 1), substantiating their liquid-like character and structural similarities in both salt regimes.

### Interactions driving high-salt phase separation of FUS and the positively charged PR_25_ peptide

The observed reentrant phase behavior provides an opportunity to shed light on the molecular processes that lead to condensate formation in the high-salt regime, and to probe whether these phenomena are the same at high and low-salt concentrations. Condensation at and below physiological salt concentrations, for example of FUS^18,19^, is mainly driven by an interplay of both electrostatics^21,61^ and hydrophobic interactions^62,63^, and can be modulated by RNA^20^ and ATP^64,65^ both of which favor and disrupt phase separation depending on their concentration. This broad mix of forces that drive and modulate phase separation at physiological salt concentrations is supported by the widespread use of charge-modifying post-translational modifications in cells to control the strength of protein–protein^66^ and protein–DNA interactions^67^, and the ability of uncharged proteins to fold and self-assemble into large complexes^68^.

From these observations, it follows that the molecular processes that lead to demixing and condensate formation in the high-salt regime are likely connected to electrostatic protein–protein interactions becoming negligible and the hydrophobic effect is heightened. Indeed, the significant drop in protein solubility upon the addition of salt, which can result in precipitation for many proteins (i.e., the salting-out effect), has been attributed to hydrophobic effects^48,69^. Thus, we reasoned that enhanced hydrophobic interactions and weakened ionic interactions might be the key drivers of protein reentrant phase separation at high-salt concentration.

**Fig. 3:**
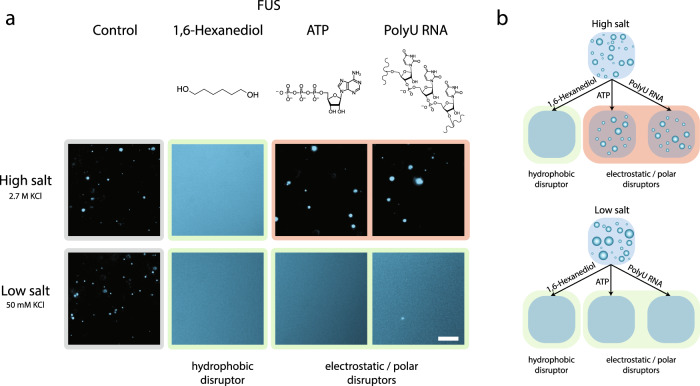
Dissolution assay of FUS condensates in the high- and low-salt regime using hydrophobic and electrostatic/polar disruptors. **a** Representative images of FUS condensates upon addition of 1,6-hexanediol, ATP, and PolyU RNA are shown. Total protein concentration was 4.5 µM and final additive concentrations were 10% 1,6-hexanediol, 1.25 mg/mL PolyU RNA, 12.5 mM ATP in 50 mM Tris-HCl (pH 7.2) at 50 mM (low salt) and 2.7 M KCl (high salt). Conditions at which the disrupters dissolved the condensates are highlighted in green and those where condensates remained intact are highlighted in red. Scale bar is 20 μm. The images are representative of the observed reproducible behavior from at least three test replicates of the respective protein/salt conditions. **b** Schematic representation of the ability for electrostatic/polar disruptor molecules ATP and PolyU RNA to dissolve condensates in the low-salt regime but not in the high-salt regime, and for the hydrophobic disruptor 1,6-hexanediol to dissolve condensates in both regimes.

To test this hypothesis, we probed the ability of pre-formed FUS condensates to dissociate when exposed to a range of additional components acting as phase separation disruptors, as shown in Fig. 3a. We selected a representative set of compounds with the ability to modulate both electrostatic interactions, such as poly-uridine (PolyU) RNA and ATP, both highly negatively charged molecules previously described to disrupt phase separation^20,64,65^, as well as 1,6-hexanediol, an aliphatic alcohol known to disrupt weak protein–protein hydrophobic interactions and selectively dissolve liquid condensates but not solid ones^70^. At low-salt concentrations, PolyU RNA, ATP, and 1,6-hexanediol were all able to dissolve FUS condensates, confirming that both hydrophobic and electrostatic interactions contribute to the stability of FUS condensates in the low-salt regime. At high-salt conditions, 1,6-hexanediol was the only disruptor that could dissolve FUS condensates, while the addition of PolyU RNA and ATP did not show any effects. These observations, summarized in Fig. 3b, suggest that reentrant high-salt phase separation of proteins is indeed primarily a hydrophobically driven process where electrostatics are screened out.

**Fig. 4:**
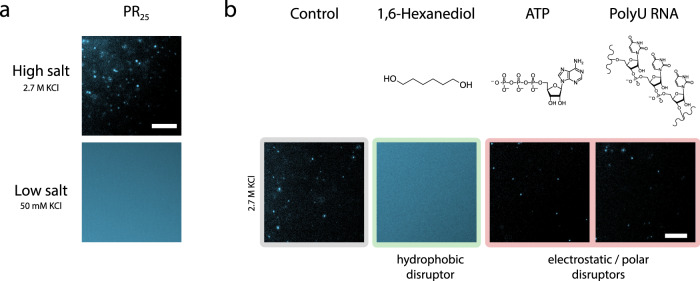
Phase separation and disruptor-mediated dissolution behavior of the PR_25_ peptide at high- and low-salt concentrations. **a** Representative images of PR_25_ at 50 mM (low salt) and 2.7 M KCl (high salt) in 50 mM Tris-HCl (pH 7.2). The unlabeled peptide was mixed with a small amount of the same peptide labeled with AlexaFluor546; total peptide concentration was 72 μM. **b** Dissolution assay of PR_25_ condensates in the high-salt regime using hydrophobic (1,6-hexanediol) and electrostatic/polar disruptors (ATP and PolyU RNA). Final peptide concentration was 54 μM PR_25_ and final additive concentrations were 10% 1,6-hexanediol, 1.25 mg/mL PolyU RNA, 12.5 mM ATP in 2.7 M KCl, 50 mM Tris-HCl (pH 7.2). Conditions at which the disruptors dissolved the condensates are highlighted in green and those where condensates remained intact are highlighted in red. Only 1,6-hexanediol dissolves PR_25_ condensates at high salt. Scale bars in all images are 20 μm. In both panels, the images are representative of the observed reproducible behavior from at least three test replicates of the respective protein/salt conditions.

To understand how modulation of salt concentration impacts phase behavior more generally, we probed the response of the highly positively charged PR_25_ peptide, which is formed by 25 repeats of the proline–arginine dipeptide (Fig. 4a). Unlike FUS, PR_25_ does not exhibit LLPS at low-salt concentrations because, in this regime, its homotypic interactions are dominated by the Arg–Arg repulsion; instead PR_25_ requires the addition of co-factor polyanions such as RNA to form complex coacervates at physiological salt^71,72^. However, at KCl concentrations of 2.7 M, we find that PR_25_ undergoes LLPS on its own (Fig. 4a). Like FUS in the high-salt regime, 1,6-hexanediol could dissolve these high-salt PR_25_ condensates, while the addition of PolyU RNA and ATP did not elicit any observable effects (Fig. 4b). These results suggest that phase separation of PR_25_ at high salt is also driven by hydrophobic interactions. Notably, this type of phase transition is not an example of reentrant phase behavior, yet it provides an additional demonstration of the occurrence of homotypical driven LLPS at high salt, enabled by the screening of electrostatic interactions, in this case, repulsion among Arg residues, and strengthening of non-charged interactions.

### Hofmeister effects on the phase behavior of FUS and PR_25_ in the high-salt regime

**Fig. 5:**
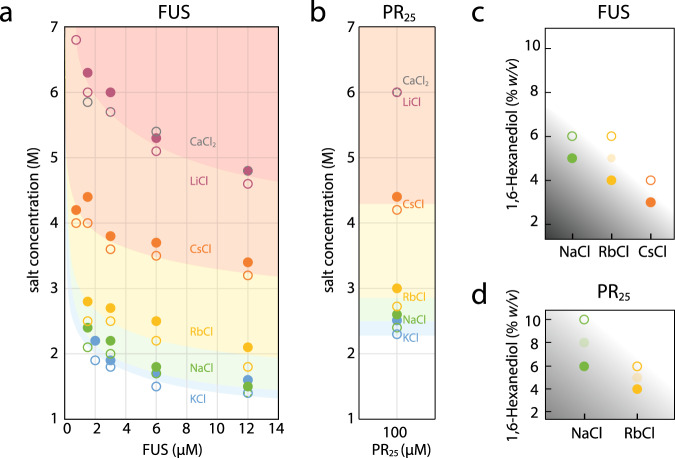
Hofmeister effect in the high-salt phase separation behavior of FUS and PR_25_. **a** Phase diagram for FUS as a function of salt concentration of various salts of the Hofmeister series. Open circles indicate cases where phase separation did not occur, closed circles indicate where phase separation did occur. Each curve depicts the apparent phase boundary for the particular salt named next to it and is only meant as a guide for the eyes. Even at the saturation concentration of CaCl_2_ (gray), the hydrophobic effect is weakened to the extent such that phase separation cannot occur, indicated by the presence of open circles and absence of closed ones in the phase diagram. **b** Phase behavior of PR_25_ as a function of ionic strength of various salts. The trend is consistent with panel **a**. **c** Comparison of the amount of 1,6-hexanediol required to dissolve FUS condensates in solutions of various salts along with the Hofmeister series. In each solution, the final salt concentration was 4 M, and the final FUS concentration was 2 μM. Partially shaded circles represent conditions where the number of condensates was visibly reduced, but the condensates were not fully dissolved. **d** Comparison of the amount of 1,6-hexanediol required to dissolve PR_25_ as a function of salts along the Hofmeister series. The final salt concentration at each point was 4 M and the PR_25_ concentration was 100 μM.

To explore further the role of hydrophobicity in phase separation in the high-salt regime, we systematically varied the chemical nature of the salts used along with the Hofmeister series^73,74^, as was previously done for the low-complexity domain of FUS at lower-salt concentrations^62^. The Hofmeister series is ordered based on the ability of ions to reduce the solubility of hydrophobic molecules in water^75^. Salts later in the Hofmeister series (e.g., Ca^2+^) increase the solubility of proteins in solution by effectively weakening the strength of hydrophobic interactions, as compared to salts earlier in the series (e.g., K^+^), which strengthen hydrophobic interactions. Hence, for a phase separation process mediated by hydrophobic interactions, higher salt concentrations should be required to induce phase separation when ascending the Hofmeister series. To test this hypothesis, we mapped out the phase behavior of FUS and PR_25_ in the high-salt regime using chloride salts of various Hofmeister series cations (Fig. 5a, b). The data show that the phase boundary shifts to higher salt concentrations in the order as given by the most agreed upon ordering of the series^75^ (i.e., K^+^, Na^+^, Rb^+^, Cs^+^, Li^+^, Ca^2+^); hence, condensate formation is disfavored with salts later in the series, as would be expected for a hydrophobically driven process. Similarly, for both FUS and PR_25_, less 1,6-hexanediol is required to dissolve condensates formed in solutions containing salts later in the Hofmeister series (Fig. 5c, d). These observations support the hypothesis that the formation of FUS and PR_25_ condensates at high-salt concentration is driven by hydrophobic interactions, and is thus of a different nature to the transition observed at low-salt concentrations, which has a significant electrostatic contribution. Our results also demonstrate that, analogous to the salting-out effect, salts across the Hofmeister series have a different impact on modulating the solubility limit of phase-separating proteins, and in shifting the boundary of immiscibility that determines phase separation.

### Molecular dynamics simulations of reentrant protein phase behavior

Next, we investigated the origin of the molecular driving forces behind reentrant protein phase behavior by developing a multiscale modeling approach that combines molecular dynamics (MD) simulations at two complementary levels of resolution: atomistic simulations of amino acid pairs and residue-resolution coarse-grained simulations of protein condensates (Simulation methods). This combination of simulations at two levels of resolution allows us to investigate how the relative contributions of electrostatic and hydrophobic interactions among atomistic amino acid pairs change as a function of salt, and subsequently to determine if such changes are consistent with protein reentrant phase behavior.

### Potential of mean force calculations of amino acid pairs as a function of salt

To this end, we first performed a set of all-atom umbrella sampling MD simulations of amino acid pairs combining residues with different chemical makeups (i.e., charge, polar atoms, *sp*^*2*^-hybridized atoms, and aromatic groups) using the state-of-the-art AMBERff03ws force field^76^ and explicit solvent and ions. From these simulations, we extract the potential of mean force (PMF) as a function of the center-of-mass (COM) distance between amino acids at different salt concentrations. By monitoring the salt-dependent changes to the various PMF minima, we estimated how the free energy that stabilizes the bound configuration of an amino acid pair is modulated by salt and the chemical composition of the pair. Since we are interested in probing interaction potentials up to very high-salt concentrations (i.e., from 0 to 3 M NaCl), it is important that the solvent and ion model parameters employed are well-fitted to reproduce adequately ion solubilities in water at 298 K (i.e., the absence of unphysical salt crystallization). We used the JC-SPC/E-ion/TIP4P/2005 force field, which has been optimized for that purpose, and verified that the correct ion solubilities are observed^77^.

Because of their dependency on polarization, investigating atomistically the effects of salt on cation–π interactions is not trivial. Cation–π interactions at low salt involve the electrostatic attraction between the polarizable quadrupole of the π-electron cloud of an aromatic ring and a polarizing positively charged amino acid^78^. Fixed charge atomistic force fields ignore polarization effects and are, therefore, unable to properly capture cation–π interactions^79,80^. While approximations to incorporate the many-body effects of polarization in atomistic force fields exist, these require many iterations at each molecular configuration, which comes at a huge computational cost, and are not free from their own inaccuracies^81^. Furthermore, the polarizing power of the cation is screened out as the salt concentration increases, which implies that the cation–π pair is significantly polarized only at low salt. Therefore, to study cation–π interactions in the context of salt-dependent protein LLPS, we have developed a specialized model by refitting the sidechain charges of Tyr and Phe amino acids when bound to Arg or Lys to describe the post-polarized state of the pairs in the low-salt regime and assumed no polarization in the moderate and high-salt regimes, where significant cation screening is expected (Supplementary Tables 1 and 2; Simulation methods).

**Fig. 6:**
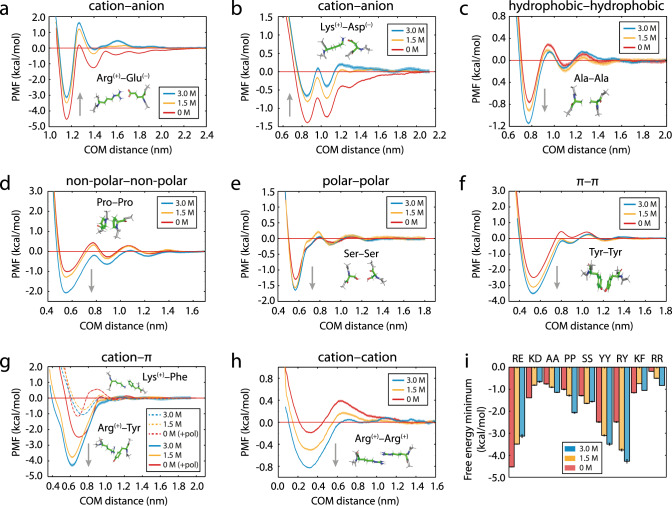
Effect of salt (0 M, 1.5 M, 3 M NaCl) on the potential of mean force (PMF) between selected amino acid pairs in explicit solvent and NaCl ions as a function of the center-of-mass (COM) distance. **a** Cation–anion (with π–π contributions), **b** cation–anion (without π–π contributions), **c** hydrophobic–hydrophobic, **d** non-polar–non-polar, **e** polar–polar, **f** π–π, **g** hybrid cation–π/π–π (Arg–Tyr, solid lines) and cation–π (Lys–Phe, dashed lines) (+pol denotes refitted Tyr/Phe parameters were employed; as described in the text and Supplementary Information), **h** Cation–cation (with π–π contribution). The second well in (**b**) emerges from the interaction of Asp with an additional H atom in the Lys sidechain, which is displaced by ~1.7 Å from the two H atoms that contribute to the first well. To evaluate (**g**), a model for the polarized cation–π systems was developed (see “Methods”). The gray arrows in each panel highlight the general shift direction of the PMF minimum as salt concentration is raised. Upward arrows show the weakening of cation–anion interactions upon increasing salt. Downward arrows show strengthening of nonionic interactions and of hybrid cation–π/π–π and cation–cation interactions when both amino acids in the pair have π–orbitals. Statistical errors, mean ± s.d., are shown as bands; obtained by bootstrapping the results from *n* = 3 independent simulations. **i** Variation in the free-energy minimum (obtained from the profiles in **a**–**h**, mean ± s.d.) with salt. One-letter amino acid codes are used to identify each pair interaction. Source data are provided as a Source Data file.

The PMF curves (Fig. 6) show that the attractive interaction energies at short intermolecular distances among oppositely charged amino acids decrease monotonically with increasing NaCl concentration (Fig. 6a, b). Conversely, those involving only uncharged residues, including polar ones, increase significantly with salt (i.e., Ala–Ala, Pro–Pro, Ser–Ser, and Tyr–Tyr) (Fig. 6c–f). Notably, interactions among a basic and an aromatic residue (e.g., Arg–Tyr or Lys–Phe, typically termed cation–π) exhibited a more complex hybrid (electrostatic–hydrophobic) behavior (Fig. 6g; discussed below), which yields a high interaction strength at both low and high salt. Interestingly, Arg–Arg (Fig. 6h) present a switch-like behavior and become attractive at high salt. A summary is given in Fig. 6i. In all cases, the strongest attractive interactions we observe, both at low and high salt, are those where the two amino acids in the pair have π–electrons (e.g., Arg–Glu, Tyr–Tyr, and Arg–Tyr/Phe) (Supplementary Fig. 2). Together, these observations support our hypothesis that, at low salt, protein condensation is stabilized by a combination of electrostatic and nonionic forces, while at high salt the hydrophobic contributions are strongest, and further suggest a dominant role of π–π interactions in both regimes.

Although interactions among cationic amino acids and aromatic residues are commonly described as mostly electrostatic—with polarization forces playing a significant role^82^—we find that they are less sensitive to screening than interactions between oppositely charged amino acids (Supplementary Fig. 2f). This finding is consistent with experimental measurements of peptide helicity showing that cation–aromatic interactions are not fully disrupted by neutral salts up to concentrations of 2.5 M^83,84^. High-level quantum chemical calculations attribute the lower sensitivity of cationic and aromatic pairs to screening to their higher orbital coordinate contributions^85^. Indeed, our results support the hypothesis interactions between basic amino acids and aromatic rings switch from cation–π electrostatic in nature at low salt to hydrophobic at high salt and are, therefore, stabilized.

Despite being universal in nature, the stabilization of hydrophobic cation–aromatic interactions at high salt has diverse molecular origins that depend on the exact chemical makeup of each pair. Lys–Phe is an example of a pair that establishes a purer cation–π bond at low salt in which no π-electrons are contributed from the Lys cation sidechain. This interaction first decreases significantly from low to moderate salt, alluding to its significant electrostatic character (Fig. 6g; dashed lines). However, contrary to solely electrostatic interaction, the Lys–Phe interaction has a shorter range and, in the high-salt regime, it regains its low-salt attractive power. This occurs because, at high salt, the π-electrons in the aromatic ring of Phe interact preferentially and more strongly with the methyl groups of Lys^86^.

Remarkably, and in contrast to the Lys–Phe cation–π pair, our PMF calculations reveal that the Arg–Tyr/Phe bond is better described as a strongly attractive hybrid cation–π/π–π interaction that increases monotonically with salt (Fig. 6g; solid lines and Supplementary Fig. 2). Both the significant strengthening with salt and the high magnitude of the Arg–Tyr/Phe interaction throughout are explained by a strong contribution of π–π bonding between the π–orbitals in the *sp*^2^-hybridized guanidinium group of Arg and the π–electrons of the aromatic rings^86–89^. This important π–π contribution was further demonstrated by performing additional PMF calculations in which the sidechain π–orbitals of Arg and Tyr were maintained in either a parallel (Supplementary Fig. 3a) or a perpendicular (t-shaped) arrangement that does not favor π–π bonding (Supplementary Fig. 3c). In contrast to the parallel Arg–Tyr pair interaction (which includes π–π stacking and, therefore, increases monotonically with increasing salt), the variation of the t-shaped Arg–Tyr interaction with salt mirrors that of Lys–Phe and is much weaker than its parallel counterpart (Supplementary Fig. 3e). Moreover, for Lys–Phe the interaction is negligibly impacted by the pair conformation (Supplementary Fig. 3b, d), which further underscores the dominant role of π–π bonding in Arg–Tyr/Phe cation–aromatic interactions, but not in Lys–Phe.

The crucial role of π–π bonds as a driving force for protein LLPS across all salt regimes, but particularly at high salt, is evident not only from the deep wells in the PMFs of *sp*^2^-hybridized cation–aromatic and aromatic–aromatic pairs (Fig. 6i) but also from the behavior of charge–charge pairs with π electrons. A remarkable example of this is the transition of the interaction between Arg–Arg from mainly an electrostatic repulsive interaction at low salt to a weak attractive π–π interaction^88,90^ in the high-salt regime (Fig. 6h). This transition occurs because once the repulsion among positive guanidinium groups is screened, the *sp*^2^-hybridized planar guanidiniums can interact via their π–orbitals. Glu, Asp, and nucleotides also have charged groups and π-orbitals, and hence, their homotypic interaction could similarly exhibit a transition from repulsion to the attraction as salt increases. Moreover, π–π bonding between Arg and nucleotides is consistent with the much higher salt-range stability observed experimentally for droplets co-assembled with poly-Arg and polynucleotides over those formed with poly-Lys and polynucleotides^89^. These unexpected results further illuminate the differences in the molecular interactions stabilizing protein condensates at high salt versus low salt and put forward Arg–Arg as an additional force that stabilizes the homotypic LLPS of PR_25_ in the high-salt regime. We, therefore, propose that at ionic strengths where charges are screened, π-driven hydrophobic interactions may sustain LLPS.

### Coarse-grained molecular dynamics simulations of FUS and PR_25_

**Fig. 7:**
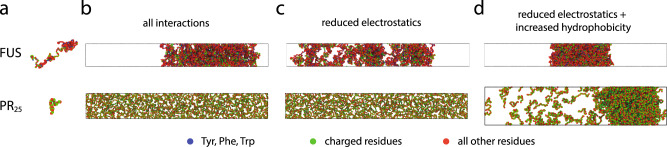
Dependence of LLPS on electrostatic versus hydrophobic forces for FUS and PR_25_ from direct coexistence simulations using a sequence-dependent protein coarse-grained model. **a** Illustration of the coarse-grained models for the different proteins with one bead representing each amino acid. Amino acids are colored according to their chemical identity (aromatics in blue, charged residues in green, all other residues in red; color code shown at the bottom). Snapshots for simulations with **b** all interactions, **c** reduced electrostatics, and **d** reduced electrostatics + increased hydrophobicity for FUS (24 proteins) and PR_25_ (400 peptides). Snapshots were rendered using Ovito^124^.

In the next step, using a coarse-grained modeling approach, we investigated whether salt-modulation of intermolecular interactions, observed atomistically, is indeed responsible for protein LLPS at high salt. For this purpose, we conducted direct coexistence simulations of tens of interacting FUS and PR_25_ polypeptide chains using a reparameterization of the amino acid resolution coarse-grained model of the Mittal group^16,91,92^, which considers sequence-dependent electrostatic and hydrophobic interactions; we developed this reparameterization to recapitulate the higher experimental LLPS propensity at low salt of FUS over its prion-like domain^93^ (Simulation methods). To investigate salt-dependent LLPS of FUS and PR_25_ (Fig. 7a), we modulated the relative contribution of electrostatic and hydrophobic interactions among amino acid pairs in the coarse-grained model, according to the salt concentration, based on our atomistic results (Fig. 6i). Consistent with our experiments at low salt, we observe LLPS for FUS (due to strong attractive electrostatic cation–anion and cation–π interactions), but not for PR_25_, which is highly enriched in positively charged amino acids that repel each other strongly in this regime (Fig. 7b, all interactions). To confirm the dependence of LLPS on electrostatic forces at low salt, we scaled down the charged–charged interactions (as suggested by our PMFs) and, as expected, observed melting of the FUS condensates (Fig. 7c; reduced electrostatics); this finding corroborates the key role of electrostatic interactions in stabilizing protein condensates at low salt. Finally, to recapitulate reentrant phase behavior at high salt, we moderately increased the strength of the hydrophobic interactions (by only 10% for FUS, including the hydrophobic attraction from the cation–π pairs at high salt, and 30% for PR_25_, as suggested by our PMF calculations), while keeping the electrostatic interactions scaled down. Indeed, we observed that this subtle enhancement of hydrophobic attraction is sufficient to yield a reentrant phase transition for FUS and induced phase separation for PR_25_ (Fig. 7d; reduced electrostatics + increased hydrophobicity). Overall, these results show that protein LLPS in the high-salt regime is driven by hydrophobic interactions with the strongest contribution coming from π–π bonds; thereby, providing a molecular explanation for our experimental observations. Our simulations further demonstrate that despite the differences in the molecular driving forces stabilizing FUS condensates at low and high salt, their molecular organization (Supplementary Fig. 4) and densities (Supplementary Table 3) are similar in both regimes. Hence, by approaching either extreme of salt, the system does in fact reenter a previously encountered phase-separated state.

## Discussion

**Fig. 8:**
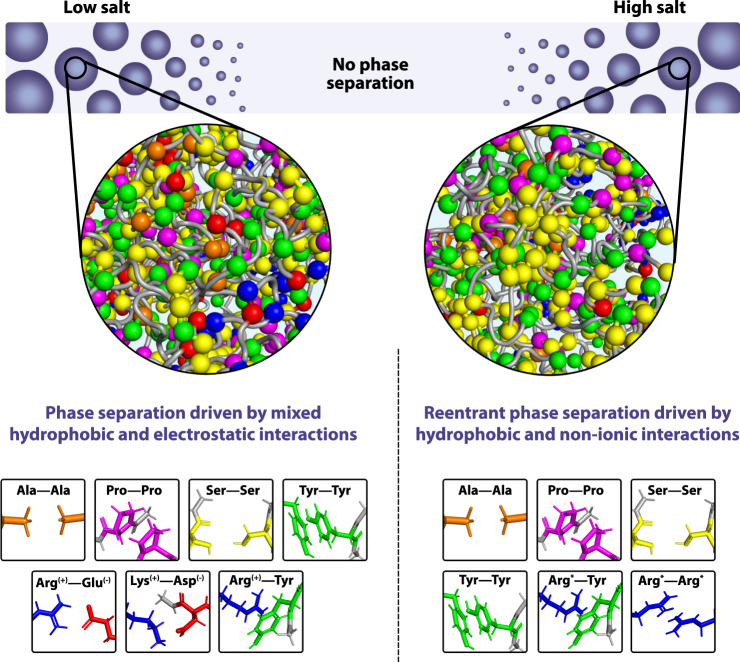
Schematic illustration of the different molecular forces that stabilize condensates in the low-salt versus the high-salt reentrant regime. While phase separation in the low-salt regime is driven by both electrostatic and hydrophobic interactions, the condensation process in the reentrant high-salt regime is governed by hydrophobic and nonionic interactions. Note: The asterisks (*) for Arg*–Try and Arg*–Arg* indicate that at high salt, charges are screened, and interactions become predominantly hydrophobic (i.e., π–π interactions).

This work demonstrates reentrant protein phase separation with salt as the control parameter. Our results show that protein molecules are driven by homotypic multivalent interactions to demix from a homogeneous phase in the limits of both low and high electrostatic screening. Previously, a different type of reentrant phase transition from the well-mixed to the phase-separated and back to the well-mixed state has been described for systems that phase-separate via heterotypic protein–RNA interactions at intermediate RNA concentrations exclusively^30–33^. Additional theoretical and experimental work has predicted the ability for reentrant phase separation of proteins, peptides, and polymers to occur as a function of pH^94^, temperature^92,95,96^, and pressure^97,98^. Our work here presents a reentrant phase transition in which proteins can phase-separate on their own in two distinct regimes in response to changes of ionic strength. Analysis of the nature of the molecular interactions implicated in biomolecular phase transitions shows that phase separation in the absence of charge screening at low-salt concentration is driven by the cooperation of electrostatic and hydrophobic interactions, while the same process at high-salt concentration is favored mainly by hydrophobic and nonionic interactions, such as interactions between Ala–Ala, Pro–Pro, Tyr–Tyr, Ser–Ser, Arg–Tyr, Arg–Arg. The latter two, as we have shown in this study, become predominantly hydrophobic in the high-salt regime and interact under those conditions predominantly through π–π bonds (Fig. 8). Importantly, we show that π–π interactions, involving both aromatic and non-aromatic residues, are dominant driving forces for LLPS in both salt regimes, but most strongly under high-salt conditions. Our work here thus highlights the role of hydrophobic and nonionic interactions as non-specific driving forces for the condensation process and therefore expands the molecular grammar of interactions governing LLPS of proteins.

The observed salt-mediated modulation of LLPS underscores the finding that the molecular driving forces for phase separation are not only dictated by the protein sequence but are also crucially sensitive to solution conditions. Solution conditions are essential to LLPS because they regulate the competition between the free-energy reduction stemming from favorable protein–protein interactions in conjunction with the release of water molecules to the bulk phase, and the entropic cost of demixing. Hence, solution conditions modulate the preferential interactions among amino acids^1,14^. In particular, our work shows that the Arg–Arg and Lys–Phe pairs exhibit a salt-dependent switch-like behavior where they transition from establishing a dominant electrostatic interaction (cation–cation repulsion and cation–π attraction, respectively) at low salt to a hydrophobic attraction at high salt stabilized by a strong π–π component (for Arg–Arg) or methyl–π interaction (for Lys–Phe). The tuneability with salt, therefore, highlights the complex interplay between specific intermolecular interactions that commit proteins to particular phases and the condensate environment, and demonstrates the ability for reentrant phase transitions to occur in biomolecular systems under conditions of varying electrostatic screening.

The finding of the existence of salt-dependent reentrant phase separation thus provides insights on the combined action of hydrophobic and nonionic interactions as molecular driving forces for the condensation process and has implications for the druggability and material properties of biomolecular condensates. In particular, aberrant phase separation of FUS, TDP-43, and Annexin A11 has been associated with neurodegenerative diseases^7^. The discovery of salt-mediated reentrant phase separation for these systems suggests a crucial feature of protein LLPS behavior that may be important when developing therapies for phase-separation implicated diseases^99^. For example, drugs that are designed to prevent or reverse-phase separation, yet induce high electrostatic screening, could in turn trigger reentrant transitions.

Moreover, pathological liquid-to-solid phase transitions in FUS, for example, are thought to be linked to the emergence of strongly-bound arrays of β-sheets, which assemble by a combination of hydrogen bonding and hydrophobic (e.g., π–π) interactions among aromatic residues within low-complexity aromatic-rich kinked segments (LARKS) that assemble into protofilaments^100^. FUS-derived peptides that contain LARKS and are devoid of charged residues interact weakly under physiological conditions (i.e., salt and temperature), as shown by their phase separation being enhanced at higher salt concentrations^33^. This observation suggests that, under physiological conditions, LARKS-driven pathological transitions are disfavored. However, in the reentrant high-salt regime, or conditions in cells that give rise to similar electrostatic screening or enhancement of hydrophobic effects (e.g., the presence of multivalent ions or other charged species and post-translational chemical modifications), our work suggests that pathological LARKS–LARKS interactions would be significantly enhanced due to strengthening of π–π interactions, thereby contributing to pathological phase transitions.

Interestingly, membraneless compartmentalization of functional protobiomolecules into protocells has been hypothesized as a potential key step in the emergence of life on earth^101,102^. Regarding this hypothesis, and mounting evidence suggesting that the salt content in early marine waters may have been very high^103,104^, likely in the molar regimes, as is still the case in hypersaline water bodies^105,106^, reentrant protein LLPS, stabilized by hydrophobic and non-ionic interactions, could have enabled the spontaneous formation of prebiotic compartments within such high salt environments.

Taken together, our study identifies salt-mediated reentrant phase separation behavior of LLPS proteins that is enabled by hydrophobic and nonionic interactions. The discovery of high-salt protein phase separation provides a compelling view of the plasticity of the molecular driving forces for protein phase separation, and emphasizes that these forces are not only defined by the amino acid sequence but also critically influenced by the condensate environment. Depending on the microenvironment, the diverse chemical makeups of amino acids allows them to engage in a multiplicity of molecular interactions (e.g., hydrophobic, electrostatic, mixed), going beyond—hence expanding—the typical one-way classification they are traditionally given (e.g., polar, hydrophobic, charged). These findings highlight the importance of considering solution conditions to which condensates are exposed when aiming at predicting, rationalizing, or modulating protein phase behavior, and when designing therapies to ameliorate phase separation-related pathologies.

## Methods

### Materials

All reagents and chemicals were purchased with the highest purity available. PolyU RNA with a molecular weight range from 800 to 1000 kDa was purchased from Sigma Aldrich as lyophilized powder and dissolved into a stock of 5 mg/mL in 50 mM Tris-HCl (pH 7.2) before use. ATP was obtained from Fisher Scientific and a 50 mM stock solution was prepared in 50 mM Tris-HCl (pH 7.2). 1,6-Hexanediol was purchased from Santa Cruz Biotechnology Inc. and a 40% (*w*/*v*) stock solution was prepared in 50 mM Tris-HCl (pH 7.2). KCl was from Fisher Scientific and all the chloride salts used in the Hofmeister series experiments were from Sigma Aldrich. The PR_25_ peptide containing 25 Pro–Arg repeats was obtained from GenScript. N-terminally labeled PR_25_ was obtained by reacting the peptide with amine-reactive AlexaFluor546 (Sigma Aldrich).

### Protein production: FUS and FUS G156E

Expression and purification of FUS wildtype and FUS G156E were adapted from Patel et al.^18^. In short, proteins were expressed in Sf9 insect cells (Expression Systems, Cat#94-001F) using the baculovirus system^107^ and produced as C-terminal EGFP fusions with an N-terminal maltose-binding protein (MBP) tag and a C-terminal hexahistidine (His_6_) tag. Cells expressing MBP-FUS-EGFP-His_6_ and MBP-FUS(G156E)-EGFP-His_6_ were harvested 72 h post infection by centrifugation at 2000 rpm for 5 min and resuspended in 50 mM Tris-HCl (pH 7.4), 1 M KCl, 5% (*w*/*v*) glycerol, 1 mM DTT, 10 mM imidazole supplemented with EDTA-free protease inhibitor cocktail set III (Calbiochem) and 0.25 U/mL benzonase (in-house, provided by the MPI-CBG protein expression facility). Cell lysis was done with an LM20 shear homogenizer (Microfluidics) at 5000 psi. The lysate was clarified by centrifugation at 16,000 rpm for 20 min. The supernatant was loaded onto Protino nickel-nitrolotriacetic acid (Ni-NTA) columns (Macherey-Nagel) with a peristaltic pump and washed with 10 column volumes of lysis buffer. Elution was done with 50 mM Tris-HCl (pH 7.4), 1 M KCl, 5% (*w*/*v*) glycerol, and 500 mM imidazole. His_6_ and MBP tags were proteolytically removed with 3C-His_6_ preScission protease (in-house, provided by the MPI-CBG protein expression facility). The mixture was incubated at room temperature for 4 h. The samples were then applied to a Superdex 200 pg 26/600 size-exclusion chromatography (SEC) column (GE Healthcare) equilibrated with 50 mM Tris-HCl (pH 7.4), 500 mM KCl, 1 mM DTT, and 5% (*w*/*v*) glycerol using a BIOCAD 60 chromatography system (Applied BioSystems). Protein fractions were pooled, concentrated with a 30,000 MWCO Amicon Ultra concentrator (Millipore), then aliquoted in tubes, flash-frozen in liquid nitrogen, and stored at −80 °C until usage.

### Protein production: TDP-43

The protein was expressed in and purified from Sf9 insect cells (Expression Systems, Cat# 94-001F) using the baculovirus system^107^. The protein was produced as a fusion protein with an N-terminal MBP tag and a C-terminal monomeric EGFP followed by a His_6_ tag. Baculovirus-infected cells expressing MBP-TDP-43-EGFP-His_6_ were harvested 72 h post infection and resuspended in lysis buffer (50 mM Tris-HCl (pH 7.4), 1 M KCl, 5% (*w*/*v*) glycerol, 10 mM imidazole, 1 mM DTT) supplemented with Complete protease inhibitor EDTA-free cocktail (Roche) and 0.25 U/mL benzonase (in-house, provided by the MPI-CBG protein expression facility). Cells were lysed with an Emulsiflex C5 high-pressure homogenizer (Avestin) at 20,000 psi. The lysate was cleared by centrifugation at 10 °C for 45 min and 25,000 rpm. The supernatant was incubated with 5 mL Ni-NTA (Qiagen) equilibrated with lysis buffer. Elution of the bound protein was done with lysis buffer supplemented with 250 mM imidazole. The MBP and His_6_ tags were removed by overnight proteolytic digest at 4 °C with 3C-His_6_ preScission protease (in-house, provided by the MPI-CBG protein expression facility). The protein was then gel-filtrated using a Superdex 200 pg 16/600 column (GE Healthcare) equilibrated with 50 mM Tris-HCl (pH 7.4), 500 mM KCl, 5% (*w*/*v*) glycerol, 1 mM DTT. Fractions containing the target protein were pooled, concentrated, aliquoted and stored at –80 °C.

### Protein production: annexin A11

The protein was expressed in and purified from *Escherichia coli* as an N-terminal His_6_-Sumo-tagged fusion protein with an ULP protease cleavage site, as previously described^28^. Briefly, the pOPINS bacterial expression vector carrying the His_6_-Sumo-tagged A11 construct was transformed into competent *E. coli* BL21(DE3) cells and expressed overnight at 25 °C in Terrific Broth (TB) autoinduction media. Cells were harvested by centrifugation for 20 min at 5000 rpm and lysed in resuspension buffer (25 mM HEPES (pH 7.4), 225 mM NaCl, 5% glycerol, 1 mM EDTA) by a high-pressure cell disruption system. The cell lysate was clarified by high-speed ultracentrifugation for 30 min at 45,000 rpm, prior to loading on a 5.0 mL Ni-Sepharose Advance (Bioserv) gravity column. After batch binding the protein for 1 h at 4 °C, the column was washed with 10 column volumes of wash buffer (25 mM HEPES (pH 7.4), 225 mM NaCl, 5% glycerol, 1 mM EDTA, and 25 mM imidazole). Protein elution was done with 25 mM HEPES (pH 7.4), 225 mM NaCl, 5% glycerol, 1 mM EDTA, and 250 mM imidazole. Purified column eluates were run on an SDS-PAGE and the fractions containing the protein were pooled, mixed with ULP protease (at a fusion protein: protease ratio of 200:1) to remove the tags. After treatment with the His_6_-tagged ULP protease and dialysis in 25 mM HEPES (pH 7.4) and 225 mM NaCl, the cleaved protein was applied onto a second Ni-Sepharose Advance column (Bioserv) to remove the His_6_-Sumo tag and the His_6_-ULP. Purified protein-containing fractions were pooled and concentrated using a Vivaspin (Generon) concentrator (30,000 MWCO). All purification steps were performed at 4 °C. The concentrated sample was aliquoted and snap-frozen using liquid nitrogen and stored at –80 °C for subsequent use. Labeling of purified A11 protein using AlexaFluor647 dye was performed following the manufacturer’s instructions (Thermo Scientific). Briefly, 2.0 mg of the protein in 25 mM HEPES, 225 mM NaCl pH 7.4 buffer was mixed with 100 µg of the dye and incubated at 4 °C for 4 h. Following incubation, the protein was applied on a Superdex 25 column (Biorad) to separate the free dye from the conjugated protein‒dye complex. The labeled protein was aliquoted, snap-frozen in liquid nitrogen, and stored at –80 °C until usage.

### Protein production: Sox2

The protein was expressed in and purified from Sf9 insect cells (Expression Systems, Cat#94-001F) using the baculovirus system^107^ and produced as a C-terminal monoGFP fusion protein with N-terminal His_6_ and MBP tags. Cells expressing His_6_-MBP-Sox2-GFP were harvested 72 h post infection and resuspended in Sox2 buffer (50 mM Bis-Tris-Propane (pH 7.5), 500 mM KCl, 5% glycerol, 1 mM DTT) supplemented with EDTA-free protease inhibitor cocktail set III (Calbiochem) and 0.25 U/mL Benzonase (in-house) and lysed by sonication. The lysate was clarified by centrifugation for 2.5 h at 13,000×*g* and 4 °C. The supernatant was applied to an Econo-Pac gravity columns (Bio-Rad) filled with amylose resin (NEB). After washing the beads with three column volumes of Sox2 buffer, the protein was eluted with Sox2 buffer supplemented with 50 mM maltose. The His_6_-MBP moiety was removed by incubation with 140 U 3C preScission protease (in-house) on a rotator for 3 h at room temperature. After concentrating of the protein to about 2.5 mL using a 50,000 MWCO Amicon Ultra concentrator (Millipore), the protein was diluted again with a solution containing 50 mM Bis-Tris-Propane pH 7.5, 5% glycerol and 1 mM DTT to a final KCl concentration of 100 mM. Next, cation exchange was performed using a HiTrap SP HP column (GE Healthcare) and 50 mM Bis-Tris-Propane pH 7.4, 5% glycerol and 1 mM DTT as a buffer with a KCl gradient ranging from 100 mM to 1 M. Fractions containing Sox2-GFP were pooled and subjected to SEC using a Superdex 200 Increase 10/300 GL column (GE Healthcare) and Sox2 storage buffer (50 mM Bis-Tris-Propane (pH 7.5), 500 mM KCl, 1 mM DTT, 5% glycerol). As before, protein-containing fractions were pooled and concentrated with a 30,000 MWCO Amicon Ultra concentrator (Millipore). The protein was then aliquoted, snap-frozen in liquid nitrogen, and stored at –80 °C until usage.

### Protein production: Brd4

The proteins was expressed in and purified from Sf9 insect cells (Expression Systems, Cat# 94-001F) using the baculovirus system^107^ and produced as an N-terminal monoGFP fusion protein with His_6_ and MBP tags. Cells expressing His_6_-MBP-GFP-Brd4 were resuspended in Brd4 buffer (50 mM Tris pH 7.5, 500 mM KCl, 5 mM EDTA, 5% glycerol) supplemented with EDTA-free protease inhibitor cocktail set III (Calbiochem) and 0.25 U/mL Benzonase (in-house) and lysed by sonication. The lysate was clarified by centrifugation for 1 h at 13,000 *g* and 4 °C. The supernatant was applied to an Econo-Pac gravity column (Bio-Rad) filled with amylose resin (NEB). After washing the beads with three column volumes of Brd4 buffer, the protein was eluted with Brd4 buffer supplemented with 50 mM maltose. The His_6_-MBP moiety was removed by incubation with 70 U 3 C preScission protease (in-house) on a rotator for 2 h at room temperature. After concentrating of the protein using a 30,000 MWCO Amicon Ultra concentrator (Millipore), the protein subjected to SEC using a Superdex 200 Increase 10/300 GL column (GE Healthcare) and Brd4 storage buffer (50 mM Tris pH 7.5, 500 mM KCl, 2 mM EDTA, 5% glycerol, 1 mM DTT). As before, protein-containing fractions were pooled and concentrated with a 30,000 MWCO Amicon Ultra concentrator (Millipore). The protein was then aliquoted, snap-frozen in liquid nitrogen, and stored at –80 °C until usage.

### Sample preparation and generation of phase diagrams

To induce phase separation, appropriate amounts of salt, water, and additives, as indicated, were added to the protein/peptide stock solutions and mixed by pipetting. In all cases the buffer contained 50 mM Tris-HCl (pH 7.2), except for Brd4 and Sox2, which contained 5 mM Tris (pH 7.5), 0.2 mM EDTA, 0.5% glycerol and 5 mM Bis-Tris-Propane (pH 7.5), 0.5% glycerol, respectively. Phase-separated samples were prepared in tubes and imaged within 1–5 min to limit any aging effects; Brd4 and Sox2 were imaged after an incubation period of 10–20 min.

Phase diagrams of FUS and PR_25_ were generated by mixing protein/peptide stocks with the respective salt solutions in 50 mM Tris-HCl (pH 7.2), as described above. The resulting sample solutions were then imaged immediately after preparation. Phase diagrams were constructed by systematically screening through conditions and assessing conditions in which a dense phase or a well-mixed state was detected. Regions close to the phase boundary were mapped out and each point on the phase diagram was tested at least three times.

### Fluorescence imaging

Imaging of FUS, FUS G156E, TDP-43, A11, and PR_25_ samples was performed on an inverted fluorescence microscope (Zeiss AxioObserver D1) equipped with a high-sensitivity camera (Evolve 512 EMCCD, Photometrics) by placing an aliquot of the sample (3 μL) on a microscope slide mounted on the microscope stage. Images were acquired using the MetaMorph acquisition software (Molecular Devices). FUS, FUS G156E, TDP-43, and PR_25_ samples were imaged using a Zeiss A-Plan 20x/0.30 NA air objective; A11 samples were imaged on a Zeiss A-Plan 100x/1.25 NA oil-immersion objective. In experiments with FUS proteins and TDP-43, an appropriate filter set for GFP detection was used (49002, Chroma Technology). Similarly, labeled PR_25_ was detected with a filter set for AlexaFluor546 fluorescence detection (49004, Chroma Technology). A11 was imaged with a filter set for AlexaFluor647 fluorescence detection (49009, Chroma Technology). Imaging of Brd4 and Sox2 was performed on a Nikon-Andor Eclipse Ti inverted spinning disc confocal microscope equipped with an Andor iXON 897 EMCCD camera and a 60x/1.2 NA water-immersion objective (Nikon). Brd4 and Sox2 samples were transferred to glass slides that were pre-prepared by first cutting a strip of double-sided tape (Scotch) into 1 × 1 cm squares which were then stuck onto the glass slides. In total, 2 µL of the sample was pipetted onto each slide and a PEGylated coverslip was used to seal the chamber in the distance of the tape. For experiments involving dissolution induced by additives, components were mixed 3:1 with 3 μL of phase-separated protein at the specified salt concentration with 1 μL of the additional component (i.e., 1,6-hexanediol, PolyU RNA, ATP). The final concentrations of protein and additional components are stated in each figure.

#### Simulation methods: PMF calculations

PMF calculations were carried out using the GROMACS simulation package (version 2019.3)^108^. Amino acids were modeled using the AMBERff03ws force field^76^. Since we are interested in probing interaction potentials at very high-salt concentrations (up to 3 M NaCl), it is very important that the solvent and ion model parameters employed are well-fitted to fairly reproduce ion solubilities in water at 298 K (i.e., an absence of unphysical salt crystallization). The JC-SPC/E-ion/TIP4P/2005 force field has been optimized for that purpose, and so it was used in this work^77^. The N- and C-terminal ends of each amino acid were capped with acetyl and N-methyl capping groups, respectively. Pairs of amino acids were oriented with their sidechains facing each other, based on the most common arrangements observed in protein structures. Dimers were immersed in a cubic box containing TIP4P/2005 water molecules (ca. 1400–3400 molecules) with a minimum distance of 1.0 nm between the dimer and the edge of the box. When necessary, some water molecules were replaced by Na^+^ and/or Cl^–^ ions to produce neutral systems. Energy minimizations (force tolerance = 500 kJ mol^–1^ nm^–1^) were performed for the neutralized systems, with positional restraints of 20,000 kJ mol^–1^ nm^–2^ applied in each dimension to all amino acid heavy-atoms. Na^+^ and Cl^–^ ions were then added to yield the desired salt concentrations (0 M, 0.15 M, 1.5 M, 3 M).

For production runs, positional restraints of 1000 kJ mol^–1^ nm^–2^ (in the directions perpendicular to the pulling direction) were used to constrain amino acid heavy-atoms. The center-of-mass (COM) distance between amino acid pairs was restrained with a harmonic umbrella potential (pulling force constant = 6000 kJ mol^−1^ nm^−2^). Bonds with hydrogens were constrained using the LINCS algorithm, permitting an integration time step of 2 fs. Periodic boundary conditions (PBC) were used during MD simulations and electrostatics were computed using Particle-Mesh Ewald summations^109^ with a Coulomb cutoff of 0.9 nm. For each concentration, ~30 windows, spaced at 0.05 nm from 0.1 to 1.6 nm, were used per amino acid pair. Each window was simulated for 10 ns. Three independent simulations were conducted for each umbrella sampling window (i.e., an aggregate simulation time of 30 ns per window). Umbrella sampling simulations were analyzed using WHAM^110^, as implemented in GROMACS. The first 1000 ps of simulations were used for equilibration and were not included in the WHAM analysis. Error analysis was performed using the Bayesian bootstrap method^111^.

### Simulation methods: cation–π charge refitting

To study the impact of salt on cation–π interactions, which are not well captured by standard atomistic force fields^79^ because they involve the polarization of the π-electron cloud^80^ of an aromatic sidechain (Tyr, Phe, Trp) due to the cationic sidechain (Lys, Arg), we refitted the charges on the Tyr and Phe sidechains when bound to Arg or Lys. We first performed constrained (i.e., backbone and capping group heavy-atoms were frozen) geometry optimizations of each dimer (Arg–Tyr, Arg–Phe, Lys–Phe) at the MP2/6-31G(d) level of theory using the Gaussian 09 program^112^. The electrostatic surface potential (ESP) was then computed for each optimized pair at HF/6-31G(d) level. Finally, the sidechain charges of Tyr and Phe were refitted based on the quantum mechanical EPSs using the RESP program in Amber (maintaining the charge symmetry in the rings)^113^.

### Coarse-grained protein model

We have implemented a reparametrized version (see ‘*Experimental validation and refinement of coarse-grained model’* below) of the sequence-dependent coarse-grained model of the Mittal group, originally developed to capture qualitatively the sequence-dependent phase behavior of proteins that undergo LLPS at physiological salt conditions (~100 mM NaCl)^16^. The model implements a resolution of one bead per amino acid and a sequence-dependent set of parameters derived top-down to approximate the single-molecule experimental radius of gyration of a wide range of intrinsically disordered proteins. Intrinsically disordered protein regions are treated as flexible polymers and globular regions as rigid bodies. Inter-residue bonds within the disordered domains are described using harmonic springs. Long-range electrostatics are modeled using a Coulombic term with Debye–Hückel electrostatic screening. Nonbonded pairwise interactions are modeled using a knowledge-based potential termed hydrophobicity scale (HPS) model that is based on one of the hydrophobicity scales for amino acids available^114^. For the globular protein domains, a 30% scaled-down set of the HPS parameters was used to account for buried amino acids. Because the model distinguishes between disordered and globular protein regions and maintains the secondary structure of globular regions, it requires an initial atomistic model for the proteins; these are described below.

### Initial atomistic models for coarse-grained simulations

We simulated the phase behavior of the full-length FUS protein (Uniprot code: K7DPS7, 526 residues, 24 proteins), the prion-like domain (PLD) of FUS for validation only (residues: 1–163, 100 proteins), and a reduced version of the PR_25_ protein (13 Arg and 12 Pro residues alternately positioned, 400 proteins). Since the structure of full-length FUS has not been resolved, we developed an atomistic model by fusing the intrinsically disordered regions with the resolved structural domains (residues from 285–371 (Protein Data Bank (PDB) code: 2LCW) and from 422–453 (PDB code: 6G99)). An initial intrinsically disordered model for PR_25_ was developed in VMD^115^.

### Coarse-grained simulation methods

To evaluate the formation of liquid condensates in the different systems, we performed direct coexistence simulations^116–118^ at constant volume and temperature. The direct coexistence method simulates the condensate and diluted phases in the same box separated by an interface. The initial simulation box was prepared by running simulations at constant temperature and a pressure of 1 bar, using the Berendsen barostat, and then enlarging the simulation box in one direction approximately four times. The simulation temperatures were chosen to be just below the correspondent critical temperatures for each system: 400 K for full-length FUS and 200 K for PR_25_. For the production runs, each system was simulated for ~2.5 μs, using a Langevin thermostat with a relaxation time of 5 ps and a time step of 10 fs^119^. The LAMMPS software MD package was used to carry out all the coarse-grained simulations^120^.

### Experimental validation and refinement of coarse-grained model

To verify that the HPS model captures qualitatively the experimental phase behavior of FUS at low salt, we first used it to compute the phase diagrams of the PLD of FUS and the full FUS protein. While experiments demonstrate a higher LLPS propensity of the full FUS protein than of the PLD^15,93^, our simulations with the original parametrization found a ~20% higher critical point for the PLD of FUS versus full FUS. The PLD is almost completely devoid of charged residues but rich in Tyr, and its LLPS has been shown experimentally to be stabilized by hydrophobic forces^62^. In contrast, full FUS contains three Arg-rich motifs, and experiments demonstrate that its LLPS at low salt is dependent on Arg–Tyr cation–π interactions^21^, and electrostatic screening inhibits LLPS (as demonstrated experimentally in this work). These findings suggested that enhanced cation–π and electrostatic interactions were required to achieve a qualitative agreement with the FUS experimental behavior. When we included an additional term in the potential energy to increase the strength of cation–π interactions at low salt, as recently proposed^121^, we qualitatively recover a higher critical temperature for full FUS versus its PLD. Our PMFs indicate that when transitioning from low to moderate salt, electrostatic interactions diminish significantly, while hydrophobic interactions remain strong, giving rise to reentrant phase behavior. However, when we tested the HPS + cation–π enhanced model in the extreme scenario of no electrostatic contribution to the potential energy and constant hydrophobicity, we observed no statistically significant difference in the critical temperature of FUS (with respect to the normal HPS + cation–π enhanced model); suggesting that this combination of parameters now underestimates the relative electrostatic contribution to the potential energy for FUS. We thus investigated the modulation of the phase diagram of full FUS in the HPS + cation–π enhanced model versus the relative electrostatic contribution to the potential energy by multiplying the Coulomb interaction by a parameter *χ* = 0, 1, 2, and 4. We found qualitatively similar phase diagrams for *χ* = 0, 1 and 2, and a 4% increase in the critical temperature for FUS with *χ* = 4; suggesting that in the HPS + cation–π enhanced model with *χ* = 0, 1 and 2 electrostatics still do not play a significant role in the phase behavior of FUS. Hence, to mimic low salt conditions, we increased the electrostatic contribution to the potential energy by a factor of four (*χ* = 4) and used this as our reference model. With this reparameterization, we recomputed the phase diagrams of full FUS and its PLD at low salt, and obtained a convincingly higher critical point for the full FUS (~15%) with respect to that of the PLD, in qualitative agreement with experimental observations^15,93^. Based on the salt-dependent trends from our PMFs, we approximate the moderate salt regime (1.5–3 M NaCl) by scaling down the strength of electrostatic interactions with respect to our reference model (i.e., we set *χ* = 2), and the high-salt regime (>3 M NaCl) by setting *χ* = 1 and increasing the hydrophobic contribution by 10–30%.

### Estimation of contact frequencies from coarse-grained simulations

The average number of protein contacts within phase-separated condensates were calculated using the MDAnalysis Python library^122,123^. Amino acids in two different proteins are in contact if they are within a cutoff distance of 0.65 nm of each other. Using this criterion, we estimated the frequency of contacts between the different domains of FUS (see Supplementary Fig. 4).

## Supplementary information

Supplementary Information

## Data Availability

Data supporting the findings of this paper are available from the corresponding authors upon reasonable request. A reporting summary for this Article is available as a Supplementary Information file. Source Data files can be accessed via the Figshare repository (10.6084/m9.figshare.13536884). The sequence of full-length FUS (UniProt accession code: K7DPS7), as well as the resolved structural domains (residues from 285–371 (PDB code: 2LCW) and from 422–453 (PDB code: 6G99)) used in atomistic models for coarse-grained simulations, are available at UniProt (https://www.uniprot.org/) and the Protein Data Bank (https://www.rcsb.org/), respectively.

## References

[1] Hyman AA, Weber CA, Jülicher F (2014). Liquid-liquid phase separation in biology. Annu. Rev. Cell Dev. Biol..

[2] Banani SF, Lee HO, Hyman AA, Rosen MK (2017). Biomolecular condensates: organizers of cellular biochemistry. Nat. Rev. Mol. Cell Biol..

[3] Shin Y, Brangwynne CP (2017). Liquid phase condensation in cell physiology and disease. Science.

[4] Welsh TJ, Shen Y, Levin A, Knowles TPJ (2018). Mechanobiology of protein droplets: force arises from disorder. Cell.

[5] Klosin A (2020). Phase separation provides a mechanism to reduce noise in cells. Science.

[6] Yoo H, Triandafillou C, Drummond DA (2019). Cellular sensing by phase separation: using the process, not just the products. J. Biol. Chem..

[7] Alberti S, Dormann D (2019). Liquid–liquid phase separation in disease. Annu. Rev. Genet..

[8] Molliex A (2015). Phase separation by low complexity domains promotes stress granule assembly and drives pathological fibrillization. Cell.

[9] Bouchard JJ (2018). Cancer mutations of the tumor suppressor SPOP disrupt the formation of active, phase-separated compartments. Mol. Cell.

[10] Berry J, Brangwynne CP, Haataja M (2018). Physical principles of intracellular organization via active and passive phase transitions. Rep. Prog. Phys..

[11] Bentley EP, Frey BB, Deniz AA (2019). Physical chemistry of cellular liquid‐phase separation. Chem. – A Eur. J..

[12] Dignon GL, Best RB, Mittal J (2020). Biomolecular phase separation: from molecular driving forces to macroscopic properties. Annu. Rev. Phys. Chem..

[13] Brangwynne CP, Tompa P, Pappu RV (2015). Polymer physics of intracellular phase transitions. Nat. Phys..

[14] Ribeiro SS, Samanta N, Ebbinghaus S, Marcos JC (2019). The synergic effect of water and biomolecules in intracellular phase separation. Nat. Rev. Chem..

[15] Wang J (2018). A molecular grammar governing the driving forces for phase separation of prion-like RNA binding proteins. Cell.

[16] Dignon GL, Zheng W, Kim YC, Best RB, Mittal J (2018). Sequence determinants of protein phase behavior from a coarse-grained model. PLoS Comput. Biol..

[17] Alberti S (2017). Phase separation in biology. Curr. Biol..

[18] Patel A (2015). A liquid-to-solid phase transition of the ALS protein FUS accelerated by disease mutation. Cell.

[19] Murakami T (2015). ALS/FTD mutation-induced phase transition of FUS liquid droplets and reversible hydrogels into irreversible hydrogels impairs RNP granule function. Neuron.

[20] Maharana S (2018). RNA buffers the phase separation behavior of prion-like RNA binding proteins. Science.

[21] Qamar S (2018). FUS phase separation is modulated by a molecular chaperone and methylation of arginine cation-π interactions. Cell.

[22] St George-Hyslop P (2018). The physiological and pathological biophysics of phase separation and gelation of RNA binding proteins in amyotrophic lateral sclerosis and fronto-temporal lobar degeneration. Brain Res..

[23] Wang A (2018). A single N‐terminal phosphomimic disrupts TDP‐43 polymerization, phase separation, and RNA splicing. EMBO J..

[24] McGurk L (2018). Poly(ADP-ribose) prevents pathological phase separation of TDP-43 by promoting liquid demixing and stress granule localization. Mol. Cell.

[25] Sabari BR (2018). Coactivator condensation at super-enhancers links phase separation and gene control. Science.

[26] Han X (2020). Roles of the BRD4 short isoform in phase separation and active gene transcription. Nat. Struct. Mol. Biol..

[27] Boija A (2018). Transcription factors activate genes through the phase-separation capacity of their activation domains. Cell.

[28] Liao YC (2019). RNA granules hitchhike on lysosomes for long-distance transport, using annexin A11 as a molecular tether. Cell.

[29] Narayanan T, Kumar A (1994). Reentrant phase transitions in multicomponent liquid mixtures. Phys. Rep..

[30] Banerjee PR, Milin AN, Moosa MM, Onuchic PL, Deniz AA (2017). Reentrant phase transition drives dynamic substructure formation in ribonucleoprotein droplets. Angew. Chem. Int. Ed..

[31] Milin AN, Deniz AA (2018). Reentrant phase transitions and non-equilibrium dynamics in membraneless organelles. Biochemistry.

[32] Choi JM, Dar F, Pappu RV (2019). LASSI: a lattice model for simulating phase transitions of multivalent proteins. PLoS Comput. Biol..

[33] Burke KA, Janke AM, Rhine CL, Fawzi NL (2015). Residue-by-residue view of in vitro FUS granules that bind the C-terminal domain of RNA polymerase II. Mol. Cell.

[34] Loo WS (2018). Reentrant phase behavior and coexistence in asymmetric block copolymer electrolytes. Soft. Matter.

[35] Zhang F (2008). Reentrant condensation of proteins in solution induced by multivalent counterions. Phys. Rev. Lett..

[36] Zhang F (2010). Universality of protein reentrant condensation in solution induced by multivalent metal ions. Proteins Struct. Funct. Bioinforma..

[37] Zhang F (2014). Reentrant condensation, liquid-liquid phase separation and crystallization in protein solutions induced by multivalent metal ions. Pure Appl. Chem..

[38] Roosen-Runge F, Heck BS, Zhang F, Kohlbacher O, Schreiber F (2013). Interplay of pH and binding of multivalent metal ions: charge inversion and reentrant condensation in protein solutions. J. Phys. Chem. B.

[39] Braun MK (2018). Reentrant phase behavior in protein solutions induced by multivalent salts: strong effect of anions Cl^–^ versus NO_3_^–^. J. Phys. Chem. B.

[40] Li T, Ci T, Chen L, Yu L, Ding J (2014). Salt-induced reentrant hydrogel of poly(ethylene glycol)–poly(lactide-co-glycolide) block copolymers. Polym. Chem..

[41] Mason BD, Zhang-van Enk J, Zhang L, Remmele RL, Zhang J (2010). Liquid-liquid phase separation of a monoclonal antibody and nonmonotonic influence of Hofmeister anions. Biophys. J..

[42] Dumetz AC, Chockla AM, Kaler EW, Lenhoff AM (2008). Protein phase behavior in aqueous solutions: crystallization, liquid-liquid phase separation. Gels, Aggreg. Biophys. J..

[43] Taratuta VG, Holschbach A, Thurston GM, Blankschtein D, Benedek GB (1990). Liquid-liquid phase separation of aqueous lysozyme solutions: effects of pH and salt identity. J. Phys. Chem..

[44] Banani SF (2016). Compositional control of phase-separated cellular bodies. Cell.

[45] Banjade, S. & Rosen, M. K. Phase transitions of multivalent proteins can promote clustering of membrane receptors. *eLife* **3**, e04123 (2014). 10.7554/eLife.04123 PMC4238058 25321392

[46] Nott TJ (2015). Phase transition of a disordered nuage protein generates environmentally responsive membraneless organelles. Mol. Cell.

[47] Elbaum-Garfinkle S (2015). The disordered P granule protein LAF-1 drives phase separation into droplets with tunable viscosity and dynamics. Proc. Natl Acad. Sci. USA.

[48] Baldwin RLHow (1996). Hofmeister ion interactions affect protein stability. Biophys. J..

[49] Pegram LM (2010). Why Hofmeister effects of many salts favor protein folding but not DNA helix formation. Proc. Natl Acad. Sci. USA.

[50] Kohn WD, Kay CM, Hodges RS (1997). Salt effects on protein stability: two-stranded α-helical coiled-coils containing inter- or intrahelical ion pairs. J. Mol. Biol..

[51] Beauchamp DL, Khajehpour M (2012). Studying salt effects on protein stability using ribonuclease t1 as a model system. Biophys. Chem..

[52] Duong-Ly, K. C. & Gabelli, S. B. Chapter seven - salting out of proteins using ammonium sulfate precipitation. In *Methods in Enzymology.* (ed. Lorsch, J.) Vol. 541, 85–94. (Academic Press, 2014). 10.1016/B978-0-12-420119-4.00007-0 24674064

[53] Arakawa T, Timasheff SN (1984). Mechanism of protein salting in and salting out by divalent cation salts: balance between hydration and salt binding. Biochemistry.

[54] Curtis RA, Prausnitz JM, Blanch HW (1998). Protein‐protein and protein‐salt interactions in aqueous protein solutions containing concentrated electrolytes. Biotechnol. Bioeng..

[55] Dumetz AC, Snellinger-O’Brien AM, Kaler EW, Lenhoff AM (2007). Patterns of protein-protein interactions in salt solutions and implications for protein crystallization. Protein Sci..

[56] Leirmo S, Harrison C, Cayley DS, Record MT, Burgess RR (1987). Replacement of potassium chloride by potassium glutamate dramatically enhances protein-DNA interactions in vitro. Biochemistry.

[57] Murdoch FE, Grunwald KAA, Gorski J (1991). Marked effects of salt on estrogen receptor binding to DNA: biologically relevant discrimination between DNA sequences. Biochemistry.

[58] Xiao B, Johnson RC, Marko JF (2010). Modulation of HU-DNA interactions by salt concentration and applied force. Nucleic Acids Res..

[59] Li J (2016). BET bromodomain inhibition promotes neurogenesis while inhibiting gliogenesis in neural progenitor cells. Stem Cell Res..

[60] Ferri ALM (2004). Sox2 deficiency causes neurodegeneration and impaired neurogenesis in the adult mouse brain. Development.

[61] Monahan Z (2017). Phosphorylation of the FUS low‐complexity domain disrupts phase separation, aggregation, and toxicity. EMBO J..

[62] Murthy AC (2019). Molecular interactions underlying liquid–liquid phase separation of the FUS low-complexity domain. Nat. Struct. Mol. Biol..

[63] Kato M (2012). Cell-free formation of RNA granules: low complexity sequence domains form dynamic fibers within hydrogels. Cell.

[64] Patel A (2017). ATP as a biological hydrotrope. Science.

[65] Kang J, Lim L, Song J (2018). ATP enhances at low concentrations but dissolves at high concentrations liquid-liquid phase separation (LLPS) of ALS/FTD-causing FUS. Biochem. Biophys. Res. Commun..

[66] Nishi H, Hashimoto K, Panchenko AR (2011). Phosphorylation in protein-protein binding: effect on stability and function. Structure.

[67] Bannister AJ, Kouzarides T (2011). Regulation of chromatin by histone modifications. Cell Res..

[68] Berne BJ, Weeks JD, Zhou R (2009). Dewetting and hydrophobic interaction in physical and biological systems. Annu. Rev. Phys. Chem..

[69] Dahal YR, Schmit JD (2018). Ion specificity and nonmonotonic protein solubility from salt entropy. Biophys. J..

[70] Kroschwald, S., Maharana, S. & Simon, A. Hexanediol: a chemical probe to investigate the material properties of membrane-less compartments. *Matters*10.19185/matters.201702000010 (2017).

[71] Boeynaems S (2017). Phase separation of C9orf72 dipeptide repeats perturbs stress granule dynamics. Mol. Cell.

[72] Boeynaems S (2019). Spontaneous driving forces give rise to protein-RNA condensates with coexisting phases and complex material properties. Proc. Natl Acad. Sci. USA.

[73] Hofmeister F (1888). Zur Lehre von der Wirkung der Salze. Arch. für Exp. Pathol. und Pharmakologie.

[74] Cacace MG, Landau EM, Ramsden JJ (1997). The Hofmeister series: salt and solvent effects on interfacial phenomena. Q. Rev. Biophys..

[75] Mazzini V, Craig VSJ (2017). What is the fundamental ion-specific series for anions and cations? Ion specificity in standard partial molar volumes of electrolytes and electrostriction in water and non-aqueous solvents. Chem. Sci..

[76] Best RB, Zheng W, Mittal J (2014). Balanced protein–water interactions improve properties of disordered proteins and non-specific protein association. J. Chem. Theory Comput..

[77] Benavides AL, Aragones JL, Vega C (2016). Consensus on the solubility of NaCl in water from computer simulations using the chemical potential route. J. Chem. Phys..

[78] Minoux H, Chipot C (1999). Cation-π interactions in proteins: can simple models provide an accurate description?. J. Am. Chem. Soc..

[79] Khan HM (2016). Improving the force field description of tyrosine–choline cation–π interactions: QM investigation of phenol–N(Me)4+ interactions. J. Chem. Theory Comput..

[80] Caldwell JW, Kollman PA (1995). Cation-.pi. interactions: nonadditive effects are critical in their accurate representation. J. Am. Chem. Soc..

[81] Demerdash O, Mao Y, Liu T, Head-Gordon M, Head-Gordon T (2017). Assessing many-body contributions to intermolecular interactions of the AMOEBA force field using energy decomposition analysis of electronic structure calculations. J. Chem. Phys..

[82] Cubero E, Luque FJ, Orozco M (1998). Is polarization important in cation-π interactions?. Proc. Natl Acad. Sci. USA.

[83] Shi Z, Olson CA, Kallenbach NR (2002). Cation-π interaction in model α-helical peptides. J. Am. Chem. Soc..

[84] Shi Z, Olson CA, Bell AJ, Kallenbach NR (2001). Stabilization of α-helix structure by polar side-chain interactions: complex salt bridges, cation-π interactions, and C-H···O H-bonds. Biopolym. - Pept. Sci. Sect..

[85] Xie N-Z, Du Q-S, Li J-X, Huang R-B (2015). Exploring strong interactions in proteins with quantum chemistry and examples of their applications in drug design. PLoS ONE.

[86] Dyson HJ, Wright PE, Scheraga HA (2006). The role of hydrophobic interactions in initiation and propagation of protein folding. Proc. Natl Acad. Sci. USA.

[87] Andrew CD (2002). Stabilizing interactions between aromatic and basic side chains in α-helical peptides and proteins. Tyrosine effects on helix circular dichroism. J. Am. Chem. Soc..

[88] Vernon, R. M. C. et al. Pi-Pi contacts are an overlooked protein feature relevant to phase separation. *eLife* **7**, e31486 (2018). 10.7554/eLife.31486 PMC5847340 29424691

[89] Fisher RS, Elbaum-Garfinkle S (2020). Tunable multiphase dynamics of arginine and lysine liquid condensates. Nat. Commun..

[90] Tesei G (2017). Self-association of a highly charged arginine-rich cell-penetrating peptide. Proc. Natl Acad. Sci. USA.

[91] Dignon GL, Zheng W, Best RB, Kim YC, Mittal J (2018). Relation between single-molecule properties and phase behavior of intrinsically disordered proteins. Proc. Natl Acad. Sci. USA.

[92] Dignon GL, Zheng W, Kim YC, Mittal J (2019). Temperature-controlled liquid-liquid phase separation of disordered proteins. ACS Cent. Sci..

[93] Kang J, Lim L, Lu Y, Song J (2019). A unified mechanism for LLPS of ALS/FTLD-causing FUS as well as its modulation by ATP and oligonucleic acids. PLoS Biol..

[94] Adame-Arana, O., Weber, C. A., Zaburdaev, V., Prost, J. & Jülicher, F. Liquid phase separation controlled by pH. *Biophys J.* **119**, 1590–1605 (2020). 10.1016/j.bpj.2020.07.044 PMC7642337 33010236

[95] Ruff KM, Roberts S, Chilkoti A, Pappu RV (2018). Advances in understanding stimulus-responsive phase behavior of intrinsically disordered protein polymers. J. Mol. Biol..

[96] Quiroz FG, Chilkoti A (2015). Sequence heuristics to encode phase behaviour in intrinsically disordered protein polymers. Nat. Mater..

[97] Cinar H (2019). Temperature, hydrostatic pressure, and osmolyte effects on liquid–liquid phase separation in protein condensates: physical chemistry and biological implications. Chem. – A Eur. J..

[98] Cinar H, Cinar S, Chan HS, Winter R (2018). Pressure-induced dissolution and reentrant formation of condensed, liquid-liquid phase-separated elastomeric α-elastin. Chem. - A Eur. J..

[99] Wheeler, R. J. et al. Small molecules for modulating protein driven liquid-liquid phase separation in treating neurodegenerative disease. Preprint at *bioRxiv*10.1101/721001 (2019).

[100] Hughes MP (2018). Atomic structures of low-complexity protein segments reveal kinked β sheets that assemble networks. Science.

[101] Cakmak, F. P., Choi, S., Meyer, M. O., Bevilacqua, P. C. & Keating, C. D. Prebiotically-relevant low polyion multivalency can improve functionality of membraneless compartments. *Nat. Commun*. **11**, 5949 (2020). 10.1038/s41467-020-19775-w PMC7683531 33230101

[102] Hyman, T. & Brangwynne, C. In Retrospect: The Origin of Life. *Nature* **491**, 524–525 (2012).

[103] Knauth, L. P. Salinity history of the Earth’s early ocean. *Nature* **395**, 554–555 (1998). 10.1038/26879 11542867

[104] Knauth, L. P. Temperature and salinity history of the Precambrian ocean: implications for the course of microbial evolution. In Geobiology: Objectives, concepts, perspectives *Elsevier* 53–69 (2005).

[105] Meybeck, M. Global distribution of lakes. In Physics and chemistry of lakes. *Springer* 1–35 (1995).

[106] Pérez, E. & Chebude, Y. Chemical analysis of Gaet’ale, a hypersaline pond in Danakil Depression (Ethiopia): New record for the most saline water body on Earth. *Aquatic Geochemistry* **23**, 09–117 (2017).

[107] Lemaitre RP, Bogdanova A, Borgonovo B, Woodruff JB, Drechsel DN (2019). FlexiBAC: a versatile, open-source baculovirus vector system for protein expression, secretion, and proteolytic processing. BMC Biotechnol..

[108] Pronk S (2013). GROMACS 4.5: a high-throughput and highly parallel open source molecular simulation toolkit. Bioinformatics.

[109] Essmann U (1995). A smooth particle mesh Ewald method. J. Chem. Phys..

[110] Kumar S, Rosenberg JM, Bouzida D, Swendsen RH, Kollman PA (1992). THE weighted histogram analysis method for free‐energy calculations on biomolecules. I. The method. J. Comput. Chem..

[111] Hub JS, De Groot BL, Van Der Spoel D (2010). G-whams-a free weighted histogram analysis implementation including robust error and autocorrelation estimates. J. Chem. Theory Comput..

[112] Frisch, M. J. et al. *Gaussian 09. Revision D.01* (Gaussian, Inc., 2013).

[113] Bayly CI, Cieplak P, Cornell WD, Kollman PA (1993). A well-behaved electrostatic potential based method using charge restraints for deriving atomic charges: the RESP model. J. Phys. Chem..

[114] Kapcha LH, Rossky PJ (2014). A simple atomic-level hydrophobicity scale reveals protein interfacial structure. J. Mol. Biol..

[115] Humphrey W, Dalke A, Schulten K (1996). VMD: visual molecular dynamics. J. Mol. Graph..

[116] Ladd AJC, Woodcock LV (1977). Triple-point coexistence properties of the Lennard-Jones system. Chem. Phys. Lett..

[117] Espinosa JR, Sanz E, Valeriani C, Vega C (2013). On fluid-solid direct coexistence simulations: the pseudo-hard sphere model. J. Chem. Phys..

[118] García Fernández R, Abascal JLF, Vega C (2006). The melting point of ice Ih for common water models calculated from direct coexistence of the solid-liquid interface. J. Chem. Phys..

[119] Rowlinson, J. S. & Widom, B. *Molecular Theory of Capillarity* (Clarendon Press, 1984).

[120] Plimpton S (1995). Fast parallel algorithms for short-range molecular dynamics. J. Comput. Phys..

[121] Das S, Lin Y-H, Vernon RM, Forman-Kay JD, Chan HS (2020). Comparative roles of charge, π, and hydrophobic interactions in sequence-dependent phase separation of intrinsically disordered proteins.. Proc. Natl Acad. Sci. USA.

[122] Gowers, R. J. et al. MDAnalysis: A Python package for the rapid analysis of molecular dynamics simulations. In *Proceedings of the 15th Python in Science Conference* (eds Benthall S. & Rostrup S.) 98–105 (Austin, TX, SciPy, 2016).

[123] Michaud-Agrawal N, Denning EJ, Woolf TB, Beckstein O (2011). MDAnalysis: a toolkit for the analysis of molecular dynamics simulations. J. Comput. Chem..

[124] Stukowski A (2010). Visualization and analysis of atomistic simulation data with OVITO—the open visualization tool. Model. Simul. Mater. Sci. Eng..

