# Supplementary material for: Reentrant liquid condensate phase of proteins is stabilized by hydrophobic and non-ionic interactions
Source: Nat Commun. 2021 Feb 17;12:1085. doi: 10.1038/s41467-021-21181-9 (PMC7889641; doi:10.1038/s41467-021-21181-9)
Supplement: Supplementary file 1 — Supplementary Information [file 41467_2021_21181_MOESM1_ESM.pdf]

Supplementary Information

Reentrant liquid condensate phase of proteins is stabilized by hydrophobic and non-ionic interactions

Georg Krainer,[†,1] Timothy J. Welsh,[†,1] Jerelle A. Joseph,[†,2,3,4] Jorge R. Espinosa,[2,3,4] Sina Wittmann,[5,6] Ella de Csilléry,[1] Akshay Sridhar,[2,3,4] Zenon Toprakcioglu,[1] Giedre Gudiškytė,[1] Magdalena A. Czekalska,[1,7] William E. Arter,[1] Jordina Guillén-Boixet,[6] Titus M. Franzmann,[6] Seema Qamar,[8] Peter St George-Hyslop,[8,9,*] Anthony A. Hyman,[5,*] Rosana Collepardo-Guevara,[2,3,4,*] Simon Alberti,[6,*] Tuomas P.J. Knowles[1,2,*]

[1] Centre for Misfolding Diseases, Yusuf Hamied Department of Chemistry, University of Cambridge, Lensfield Road, Cambridge CB2 1EW, UK

[2] Cavendish Laboratory, Department of Physics, University of Cambridge, J J Thomson Avenue, Cambridge CB3 0HE, UK

[3] Department of Genetics, University of Cambridge, Cambridge CB2 3EH, UK

[4] Yusuf Hamied Department of Chemistry, University of Cambridge, Lensfield Road, Cambridge CB2 1EW, UK

[5] Max Planck Institute of Molecular Cell Biology and Genetics (MPI-CBG), 01307 Dresden, Germany

[6] Biotechnology Center (BIOTEC), Center for Molecular and Cellular Bioengineering (CMCB), Technische Universität Dresden, Tatzberg 47/49, 01307 Dresden, Germany

[7] Institute of Physical Chemistry, Polish Academy of Sciences, Kasprzaka 44/52 01-224 Warsaw, Poland

[8] Cambridge Institute for Medical Research, Department of Clinical Neurosciences, University of Cambridge, Cambridge CB2 0XY, UK

[9] Division of Neurology, Department of Medicine, University of Toronto and University Health Network, Toronto, Ontario M5S 3H2, Canada

* Correspondence to: Tuomas P.J. Knowles (tpjk2@cam.ac.uk), Simon Alberti (simon.alberti@tu-dresden.de), Rosana Collepardo-Guevara (rc597@cam.ac.uk), Anthony A. Hyman (hyman@mpi-cbg.de), Peter St George-Hyslop (phs22@cam.ac.uk)

† These authors contributed equally to this work

The Supplementary Information includes:

Supplementary Information for atomistic PMF calculations (Supplementary Tables 1 and 2)
Supplementary Information for condensate densities (Supplementary Table 3)
Supplementary Figures 1, 2, 3, 4

Supplementary Information for atomistic PMF calculations

Supplementary Table 1: Refitted charges for Tyr in Arg–Tyr dimer and Phe in Arg–Phe and Lys–Phe dimers. All dimers are in the parallel geometry (as shown in Figure 6g of main text and in Supplementary Figure 2 below). The original and refitted force field parameters are summarized below.

Atom in Tyr	Original charges	Refitted Tyr charges in Arg–Tyr	Atom in Phe	Original charges	Refitted Phe charges in Arg–Phe	Refitted Phe charges in Lys–Phe
**CB**	-0.051853	0.516775	**CB**	-0.09872	0.755677	0.946836
**HB1**	0.019145	-0.154966	**HB1**	0.060989	-0.209079	-0.25544
**HB2**	0.019145	-0.154966	**HB2**	0.060989	-0.209079	-0.25544
**CG**	0.112601	-0.220971	**CG**	0.021313	-0.203187	-0.387676
**CD1**	-0.183461	-0.125181	**CD1**	-0.083109	-0.273037	-0.25241
**HD1**	0.132715	0.130310	**HD1**	0.098466	0.128345	0.221075
**CE1**	-0.181823	-0.299333	**CE1**	-0.156974	0.027607	-0.211827
**HE1**	0.137303	0.202092	**HE1**	0.123731	0.095427	0.167398
**CZ**	0.206277	0.337235	**CZ**	-0.099824	-0.22782	0.052773
**OH**	-0.421233	-0.492286	**HZ**	0.114679	0.160458	0.074129
**HH**	0.329691	0.376644	**CE2**	-0.156974	0.027607	-0.211827
**CE2**	-0.181823	-0.299333	**HE2**	0.123731	0.095427	0.167398
**HE2**	0.137303	0.202092	**CD2**	-0.083109	-0.273037	-0.25241
**CD2**	-0.183461	-0.125181	**HD2**	0.098466	0.128345	0.221075
**HD2**	0.132715	0.130310				

Supplementary Table 2: Refitted charges for Tyr in Arg–Tyr and Phe in Lys–Phe for amino acid pairs in t-shaped geometries (see Supplementary Figure 3 below). The original and refitted force field parameters are summarized below.

Atom in Tyr	Original charges	Refitted Tyr charges in Arg–Tyr	Atom in Phe	Original charges	Refitted Phe charges in Lys–Phe
**CB**	-0.051853	0.662808	**CB**	-0.09872	0.963787
**HB1**	0.019145	-0.177211	**HB1**	0.060989	-0.28587
**HB2**	0.019145	-0.177211	**HB2**	0.060989	-0.28587
**CG**	0.112601	-0.612723	**CG**	0.021313	-0.323652
**CD1**	-0.183461	0.185225	**CD1**	-0.083109	-0.334344
**HD1**	0.132715	0.14026	**HD1**	0.098466	0.214518
**CE1**	-0.181823	-0.626374	**CE1**	-0.156974	-0.001983
**HE1**	0.137303	0.25684	**HE1**	0.123731	0.123162
**CZ**	0.206277	0.824476	**CZ**	-0.099824	-0.187325
**OH**	-0.421233	-0.881568	**HZ**	0.114679	0.139878
**HH**	0.329691	0.472768	**CE2**	-0.156974	-0.001983
**CE2**	-0.181823	-0.626374	**HE2**	0.123731	0.123162
**HE2**	0.137303	0.25684	**CD2**	-0.083109	-0.334344
**CD2**	-0.183461	0.185225	**HD2**	0.098466	0.214518
**HD2**	0.132715	0.14026			

Supplementary Information for condensate densities

Supplementary Table 3: Densities of FUS condensates computed using coarse-grained model parameters for different salt regimes. Statistical uncertainties are given in parentheses. n.a., not applicable.

Salt regime	Density of condensate (g/cm^3)
Low salt	0.33 (7%)
Medium salt	n.a.
High salt	0.50 (7%)*

* Sensibly higher density, which means that we have crossed the coexistence line; thus, an increase in hydrophobicity of 5% (versus 10%, as described in the main text) would have been sufficient to reenter the condensed phase.

Supplementary Figures

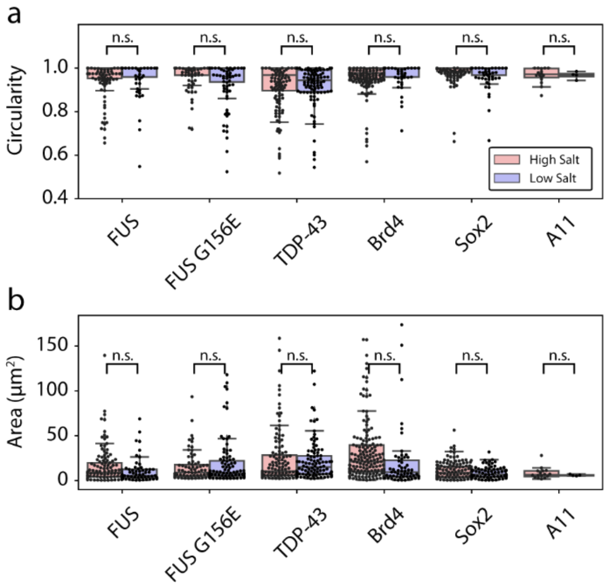

Supplementary Figure 1. Analysis of condensate circularity and condensate area in the low and high salt regimes for FUS, FUS G156E, TDP-43, Brd4, Sox2, and A11. (a) Analysis of condensate circularities. For all proteins, median circularities were >0.94. For each protein, at high salt and low salt, respectively, the median circularities were: FUS: 1.00, 1.00; FUS G156E: 1.00, 1.00; TDP-43: 0.97, 0.94; Brd4: 0.98, 1.00; Sox2: 1.00, 1.00; A11: 0.97, 0.97. Statistical analysis was performed using a two-sided *t*-test. *p*-values were found to be FUS: $p = 0.7518$; FUS G156E: $p = 0.0948$; TDP-43: $p = 0.6801$; Brd4: $p = 0.3082$; Sox2: $p = 0.1503$; A11: $p = 0.9973$. No significant difference in circularity was found between high- and low-salt condensates (n.s., not significant). **(b)** Analysis of condensate area. For each protein, at high salt and low salt, respectively, the median areas (reported in μm^2) were: FUS: 7.60, 2.95; FUS G156E: 4.60, 3.86; TDP-43: 6.70, 7.83; Brd2: 21.22, 9.43; Sox2: 8.91, 6.81; A11: 6.61, 5.87. Statistical analysis between the areas was performed using a two-sided *t*-test. *p*-values were found to be FUS: $p = 0.0645$; FUS G156E: $p = 0.2440$; TDP-43: $p = 0.3809$; Brd4: $p = 0.1710$; Sox2: $p = 0.0935$; A11: $p = 0.5040$. No significant difference in area (n.s., not significant) was found between high- and low-salt condensates. Overall, the dataset confirms the morphological similarities between condensates in the high- and low-salt regimes. Note: In each of the images the pixel resolution size is near the diffraction limit, due to the high threshold value used as a cut-off condition; hence, only condensates above the diffraction limit were used in accessing condensates circularity and size. Circularity analysis and area analysis was performed on subsections of the images shown in Fig. 2. Circularity was calculated using the formula: circularity $= 4\pi \left(\frac{\text{area}}{\text{perimeter}}\right)^2$. Image analysis was done in Fiji/ImageJ and statistical analysis was conducted in Python. For each box plot, at least two independent images at each respective protein/salt conditions were analyzed, yielding the following the number of condensates, *n*, for circularity and area analysis: $n_{\text{FUS, low salt}} = 60$, $n_{\text{FUS, high salt}} = 119$, $n_{\text{FUS G156E, low salt}} = 95$; $n_{\text{FUS G156E, high salt}} = 75$; $n_{\text{TDP-43, low salt}} = 100$; $n_{\text{TDP-43, high salt}} = 130$; $n_{\text{Brd4, low salt}} = 57$; $n_{\text{Brd4, high salt}} = 162$; $n_{\text{Sox2, low salt}} = 75$; $n_{\text{Sox2, high salt}} = 139$; $n_{\text{A11, low salt}} = 4$; $n_{\text{A11, high salt}} = 16$. In both plots, boxes extend from the 25th to 75th percentiles, with a line at the median. Whiskers span 1.5× the interquartile range. Source data are provided as a Source Data file.

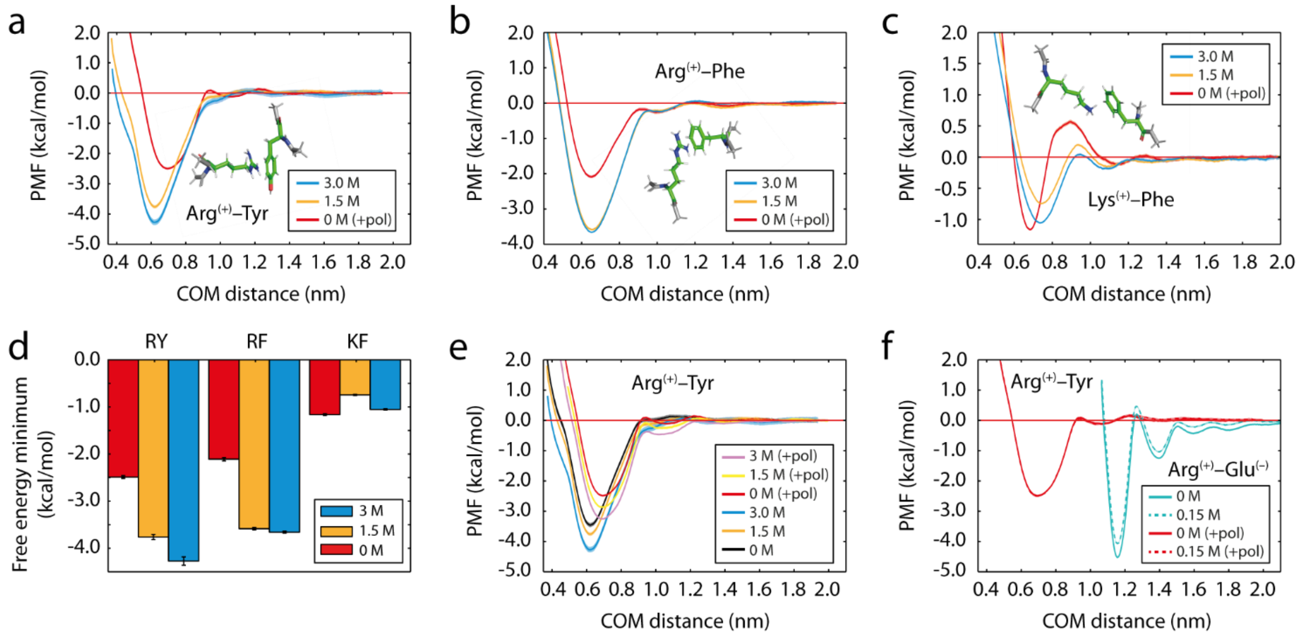

Supplementary Figure 2. Effect of salt on the potential of mean force (PMF) between basic and aromatic amino acid pairs in explicit solvent and NaCl ions as a function of the center-of-mass (COM) distance. In the plots, +pol denotes refitted Tyr/Phe parameters were employed (as described above and summarized in Supplementary Table 1). **(a)** Arg–Tyr (as in main text). **(b)** Arg–Phe. **(c)** Lys–Phe (as in main text). Statistical errors, mean ± s.d, are shown as bands; obtained by bootstrapping the results from *n* = 3 independent simulations. **(d)** Variation in the free energy minimum (obtained from the profiles in a–c, mean ± s.d.) with salt. One-letter amino acid codes are used to identify each pair interaction. **(e)** Comparison of Arg–Tyr interaction strength computed using the original force field Tyr parameters with those calculated using the modified Tyr parameters (i.e., obtained from the charge refitting procedure). **(f)** Arg–Tyr versus Arg–Glu interaction at low salt concentrations; plots reveal differences in sensitivity to electrostatic screening and variations in the interaction ranges. Source data are provided as a Source Data file.

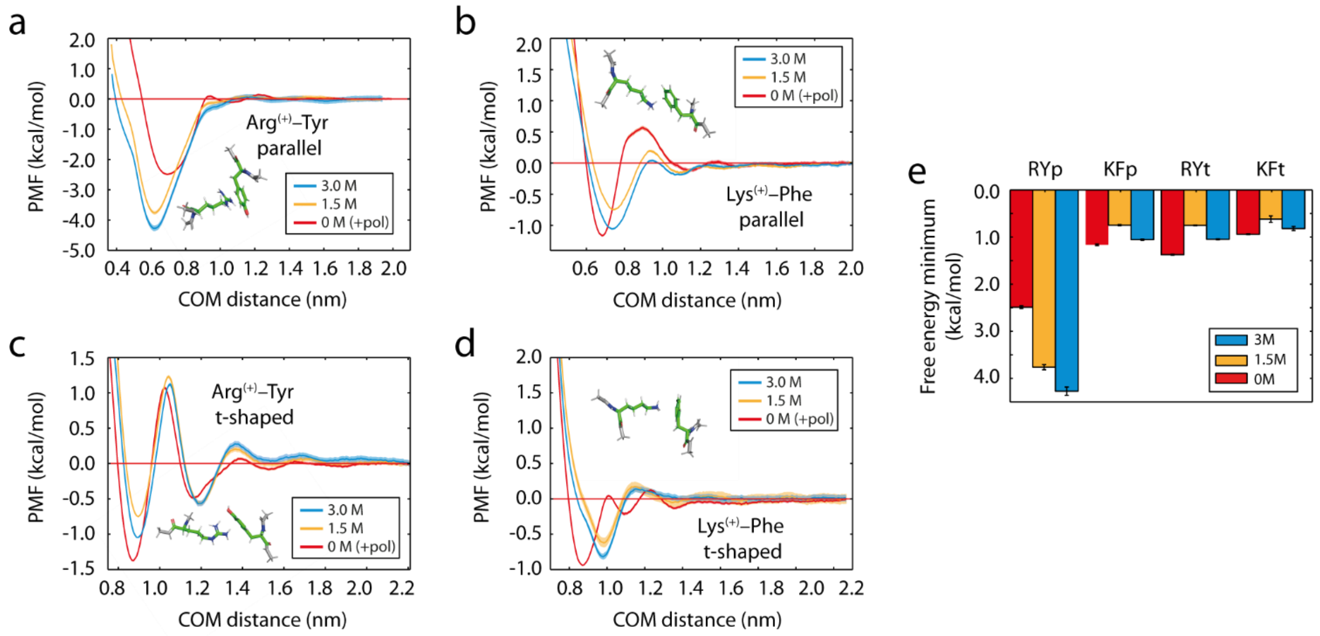

Supplementary Figure 3. Effects of geometry (parallel versus t-shaped) on the potential of mean force (PMF) between basic and aromatic amino acid pairs, computed at different salt concentrations, as a function of the center-of-mass (COM) distance. In the plots, +pol denotes refitted Tyr/Phe parameters were employed (as described above and summarized in Supplementary Tables 1 and 2). **(a)** Arg–Tyr (parallel; as in the main text). **(b)** Lys–Phe (parallel; as in the main text). **(c)** Arg–Tyr (t-shaped). **(d)** Lys–Phe (t-shaped). Statistical errors, mean ± s.d., are shown as bands; obtained by bootstrapping the results from n = 3 independent simulations. **(e)** Variation in the free energy minimum (obtained from the profiles in a–d, mean ± s.d.) with salt. Upper-case one-letter amino acid codes identify each pair interaction; lower-case p and t denote parallel and t-shaped geometries, respectively. Source data are provided as a Source Data file.

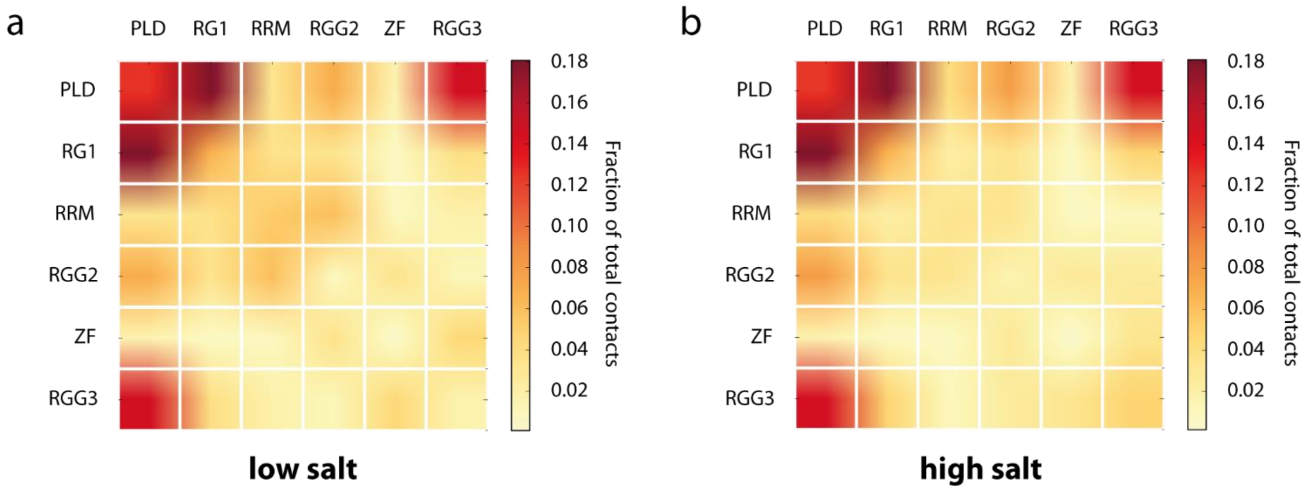

Supplementary Figure 4. Frequency of contacts between FUS domains within condensates at (a) low and (b) high salt. The FUS domains are as follows: prion-like domain (PLD: residues 1–165), arginine–glycine–glycine rich regions (RGG1: residues 166–267; RGG2: residues 371–421; RGG3: residues 454–526), RNA recognition motif (RRM: residues 282–370), and zinc finger (ZF: residues 422–453).
